# Super‐MoCo‐MoDL: A combined super-resolution and motion-corrected undersampled deep-learning reconstruction framework for three-dimensional whole-heart cardiac magnetic resonance imaging

**DOI:** 10.1016/j.jocmr.2025.101990

**Published:** 2025-11-19

**Authors:** Andrew Phair, Simon J. Littlewood, Anastasia Fotaki, Thomas J. Fletcher, Lina Felsner, Won-Yong Kim, Claudia Prieto, René Botnar

**Affiliations:** a School of Biomedical Engineering and Imaging Sciences, King’s College London, London, United Kingdom; b Department of Electrical and Electronic Engineering, Imperial College London, London, United Kingdom; c Institute for Computational Imaging and AI in Medicine, Technical University of Munich, Munich, Germany; d Department of Cardiology, Aarhus University Hospital, Aarhus N, Denmark; e Department of Clinical Medicine, Aarhus University, Aarhus, Denmark; f School of Engineering, Pontificia Universidad Católica de Chile, Santiago, Chile; g Millennium Institute for Intelligent Healthcare Engineering, Santiago, Chile; h Institute for Biological and Medical Engineering, Pontificia Universidad Católica de Chile, Santiago, Chile; i Institute for Advanced Study, Technical University of Munich, Munich, Germany

**Keywords:** Cardiac MRI, Model-based reconstruction, Motion correction, Super resolution, coronary magnetic resonance angiography (CMRA), coronary artery disease

## Abstract

**Background:**

Cardiac magnetic resonance (CMR) is a well-established imaging modality for the assessment of cardiovascular diseases. However, attainable image resolution remains lower than that of X-ray computed tomography (CT) due to long scan times and the need for respiratory motion correction. In this work, we combine a previously proposed motion-corrected model-based deep-learning reconstruction for undersampled three-dimensional (3D) whole-heart CMR with data-consistent super-resolution to enable high-resolution 3D whole-heart CMR from significantly shortened scans.

**Methods:**

Our proposed framework, Super motion-corrected (MoCo) model-based deep-learning (MoDL), utilizes two neural networks; the first estimates non-rigid respiratory motion from zero-padded and zero-filled bin images, the second applies these fields in an iterative motion-corrected model-based alternating direction method of multipliers (alternating direction method of multipliers) reconstruction which alternates between applying a super-resolving U-Net and imposing data-consistency in the acquired center of k-space. The framework was trained using 156 isotropic-resolution free-breathing 3D datasets. It was subsequently applied to prospective anisotropic low-resolution free-breathing 3D data acquired in a cohort of congenital heart disease (CHD) patients, and to prospective undersampled and low-resolution data acquired in a cohort of patients with suspected coronary artery disease (CAD).

**Results:**

Isotropic resolution whole-heart 3D images were reconstructed from ∼ 0.8- and ∼ 2.1-minute scans, for CHD patients at 1.5-mm resolution and suspected-CAD patients at 0.9-mm resolution, respectively, representing an overall scan acceleration of ∼ 18-fold in each case. Visual inspection, expert image quality scores and rankings, and quantitative vessel sharpness measurements demonstrated that the Super-MoCo-MoDL reconstructions produced sharp high-quality images that were comparable with high-resolution acquisitions. For patients with suspected CAD, comparison was made with computed tomography coronary angiography (CTCA), demonstrating that coronary plaque visualization was possible with the Super-MoCo-MoDL technique.

**Conclusion:**

Super-MoCo-MoDL is able to reconstruct high-resolution 3D whole-heart images from low-resolution and undersampled anisotropic acquisitions.

## Graphical abstract

**Figure:**
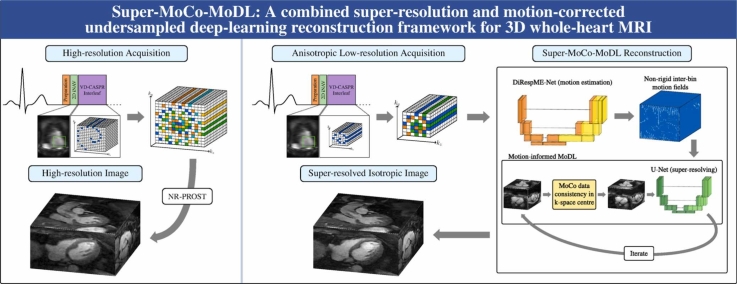

## 1 Introduction

Cardiovascular magnetic resonance (CMR) is well established as a non-invasive and ionizing-radiation-free imaging modality that can provide high-quality images with excellent soft-tissue contrast for cardiovascular disease assessment, diagnosis, and treatment planning [1], [2]. However, CMR has traditionally been limited by poor respiratory scan efficiency and the fundamentally slow nature of magnetic resonance imaging (MRI) data acquisition. Typically, a stack of two-dimensional (2D) slices of the heart are acquired during multiple breath-holds, or, if three-dimensional (3D) whole-heart coverage is required, diaphragmatic navigator gating is used to gate the acquisition to a small quiescent respiratory phase, resulting in long and unpredictable scan times[3].

Image navigators (iNAVs) have been proposed to alleviate the inefficiencies of diaphragmatic navigator gating, such as unpredictable scan times and the need for a motion model [4], [5], [6], [7], [8], [9], [10]. They allow respiratory motion to be estimated and corrected for, either via k-space phase modulations for translational correction [4], [5], [6], [7], or by also fully integrating non-rigid respiratory motion fields (estimated from intermediate respiratory-bin images) into the reconstruction pipeline [8], [9], [10], [11], [12]. Thus, 100% respiratory scan efficiency and predictable scan times can be achieved. Using iNAVs, whole-heart CMR at anisotropic 1 mm × 1 mm × 2 mm spatial resolution, for example, has scan times of ∼ 10 min [8], [11], and thus further acceleration is critical to enable whole-heart CMR with even higher isotropic resolution.

To this end, advanced reconstruction methods which exploit parallel imaging, compressed sensing, and low-rank properties have been proposed to accelerate whole-heart CMR, allowing artifact-free images to be obtained from undersampled k-space data [12], [13], [14], [15], [16], [17], [18]. However, the reconstruction times of these methods are often long, rendering them clinically infeasible.

More recently, deep-learning techniques have emerged as a promising approach for achieving accelerated CMR with fast reconstruction times [19], [20], and various methods have been proposed for high-resolution 3D whole-heart CMR applications [21], [22], [23], [24], [25], [26], [27], [28], [29], [30]. In deep learning, the parameters of deep neural networks, which are used as part of the reconstruction framework, are typically learnt via an iterative training procedure, with the subsequent application of these networks exhibiting great computational acceleration relative to their conventional counterparts. Strategies proposed for deep-learning-based CMR reconstruction include those that operate in k-space to recover missing samples [21], those that operate in image space to remove noise and undersampling artifacts [22], [23], and those that adopt an unrolled architecture [31], [32], enforcing consistency with the acquired data in an alternating iterative reconstruction [24], [25], [26], [27], [28], [29], [30]. In particular, the MoCo-MoDL (motion-corrected model-based deep-learning) technique [28], [29], [30] utilized the unrolled MoDL [31] framework while also accounting for respiratory motion by incorporating diffeomorphic non-rigid motion fields, estimated by a second deep neural network [33], [34], [35], directly into the encoding operator of the data-consistency step.

As an alternative to acquiring at the target resolution with an undersampled k-space, reducing the acquired number of k-space samples, and hence the scan time, can be achieved by fully sampling at a lower image resolution. The task of increasing the resolution from the lower acquired resolution to the higher target resolution, so-called super-resolving, is then passed to the reconstruction algorithm.

Since MRI data is acquired in the Fourier domain, sub-pixel in-plane shifts correspond to phase modulations of the acquired k-space data, and no genuine inter-pixel information is available [36], [37], [38]. Thus, prior to the development of machine-learning techniques, super-resolution (SR) in MRI was limited to through-plane applications utilizing overlapping or rotated imaging slices [39], [40], [41], [42], [43], or in-plane techniques which utilized bespoke spatially selective [44], [45] or sub-pixel-encoded [46], [47] sequences.

Conversely, learning-based methods are able to learn the properties of natural images or high-resolution MRI images and utilize these in the super-resolving process [48]. As an example, sparse-representation techniques have been proposed whereby paired dictionaries of high-resolution and low-resolution bases learnt from high-resolution-image training sets can be used to construct a high-resolution image from the sparse coefficients of a low-resolution image [49], [50]. Such ideas have been applied to achieve in-plane SR in MRI with standard acquisition sequences [51], [52], [53].

In recent years, a multitude of deep-neural-network-based SR approaches have been proposed in the field of computer vision [54], [55], [56], [57] and subsequently adapted for MRI applications [58], [59], [60], [61], [62], [63], [64], [65], [66], [67], [68], [69], [70], [71], [72], [73], [74], [75], [76], [77], [78]. These methods typically utilize convolutional neural networks to learn the relationship between low- and high-resolution images, with the low-resolution image given as input to the network and the high-resolution image returned as output. Various frameworks and network architectures have been proposed, including U-Nets [61], [62], [63], [64], residual networks [63], [64], [65], [70], [71], [72], [73], generative adversarial networks (GANs) [67], [68], [69], [70], [71], vision transformers [74], diffusion models, [75] and implicit neural representations (INRs) [75], [76], [77], [78].

In cases where deep-learning-based SR has been applied to 3D whole-heart CMR [64], [65], [66], [67], [68], the various SR networks are applied directly to a low-resolution image in an analogous way to the noise- and undersampling-artifact-removing networks previously discussed for the undersampled case [22], [23]. As such, data consistency is not explicitly guaranteed, and non-rigid motion correction is not incorporated into the super-resolving section of the reconstruction pipeline.

In this work, we propose to adapt the MoCo-MoDL method to obtain a super-resolved isotropic-resolution whole-heart 3D CMR reconstruction framework which, unlike existing SR methods in CMR, achieves 100% respiratory scan efficiency, incorporates non-rigid respiratory motion into the reconstruction, and ensures the output 3D images are consistent with the acquired data. Our proposed framework, Super-MoCo-MoDL, additionally allows low-resolution acquisitions to be acquired with k-space undersampling; both the SR and undersampled reconstruction are handled simultaneously by the framework. The framework is trained using 156 high-resolution scans for two undersampling/low-resolution schemes: fully sampled acquisitions with 4 × 4 SR in the phase-encoding plane, and 4.5-fold undersampled acquisitions with 2 × 2 SR in the phase-encoding plane. The trained framework is subsequently applied to prospective low-resolution data acquired with each of the schemes, enabling the reconstruction of 3D whole-heart images with comparable quality and sharpness to prospective high-resolution acquisitions.

## 2 Method

### 2.1 Acquisition sequences

Data were acquired using free-breathing whole-heart ECG(electrocardiogram)-triggered balanced steady-state free precession (bSSFP) sequences, as depicted in Supplementary File: Section 1. A VD-CASPR (variable density Cartesian acquisition with spiral profile order) trajectory [13], [79], which consists of a Cartesian spiral-like (in the *k*_*y*_*k*_*z*_-plane) set of fully sampled *k*_*x*_-readouts each acquisition window, was utilized. A 2D coronal iNAV [4] was also acquired each heartbeat for the estimation of in-plane translational respiratory motion.

A single-contrast version of the sequence [13] was used for all prospective low-resolution acquisitions and some high-resolution acquisitions. In this version, the preparation pulses consisted of a *T*_2_-preparation (*T*_2_-prep) pulse and a fat saturation pulse.

The training set was expanded through the inclusion of odd-heartbeat data from high-resolution scans which were acquired with two different BOOST [80] (bright-blood and black-blood phase sensitive) sequences: MTC-BOOST [81] (magnetization transfer contrast) and i*T*_2_-prep-BOOST [82] (interleaved *T*_2_-preparation). These sequences adhere to the general acquisition framework, but alternate preparation pulses such that bright-blood images are obtained from odd-heartbeat data while black-blood images are obtained from the subtraction of even-heartbeat data from odd-heartbeat data. In MTC-BOOST, the preparation for odd-heartbeat acquisitions consisted of an MT-preparation pulse followed by an inversion recovery (IR) pulse, and for even heartbeats consisted of an MT-preparation pulse followed by a fat saturation pulse. In i*T*_2_-prep-BOOST, the preparation for odd-heartbeat acquisitions consisted of a *T*_2_-prep pulse followed by an IR pulse; for even heartbeats, a single fat saturation pulse was applied.

### 2.2 Super-MoCo-MoDL reconstruction framework

**Fig. 1:**
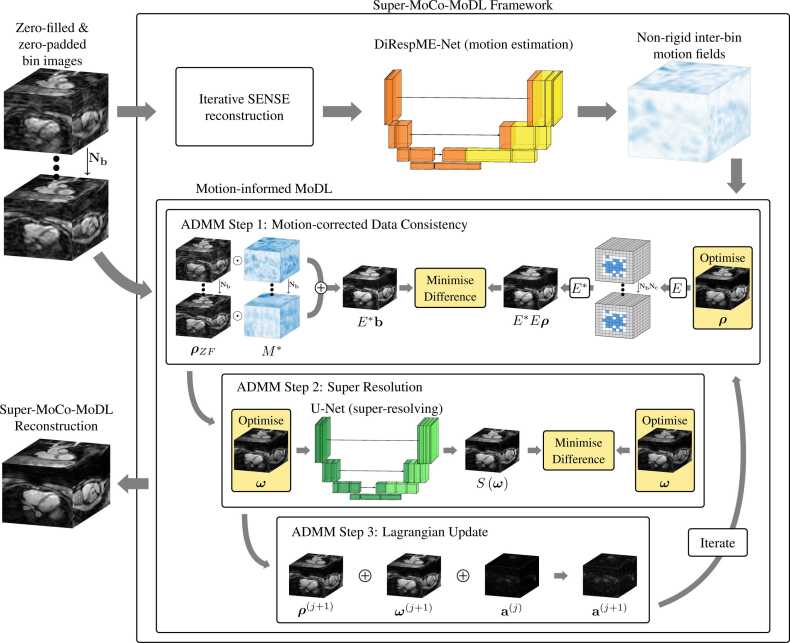
Schematic of Super-MoCo-MoDL, the super-resolving motion-corrected model-based deep-learning reconstruction framework. Zero-filled and zero-padded respiratory-bin images (or image patches) are passed through an iterative SENSE reconstruction to generate de-noised auxiliary bin images which form the input to the DiRespME-Net motion estimation network. The output non-rigid respiratory motion fields are then used together with the zero-filled images in an ADMM iteration which reconstructs a super-resolved image by solving the minimization problem expressed in Eq. (4). The ⊙ operator represents the non-rigid warping operation. *ADMM* alternating direction method of multipliers, *DiRespME-Net* diffeomorphic respiratory motion estimation network, *SENSE* sensitivity encoding

A schematic of the Super-MoCo-MoDL reconstruction framework is presented in Fig. 1. Given the input of zero-filled respiratory-bin images (or image patches) at the desired high-resolution array size, the framework estimates non-rigid motion fields between respiratory bins and then utilizes these fields in a model-based super-resolving iterative reconstruction. Each stage of the reconstruction, including the preparation of low-resolution data, the splitting of images into patches, the non-rigid motion estimation and the iterative reconstruction, is described in detail below.

#### 2.2.1 Preparation of prospective low-resolution data

Initially, the acquired low-resolution k-space data were processed to generate zero-filled respiratory-bin images with an array size matching that of the desired high-resolution 3D images. Thus, throughout the Super-MoCo-MoDL framework, low- and high-resolution images contain the same number of voxels and we consider the SR task to be one of regaining the high-frequency information lost in low-resolution acquisitions.

A respiratory-motion position for each heartbeat was estimated in each of the foot-head (*x*) and left-right (*y*) directions using the acquired iNAV images. A phase shift in k-space, corresponding to an *xy*-shift in image-space, was applied to each k-space readout in order to align the position of the heart across all acquired data. The translational-motion-corrected k-space data were then sorted into four equally populated respiratory bins using the foot-head motion signal and soft-gating [11], [83].

We now define an encoding operator, *E*, which models the data acquisition, such that, when *E* is applied to the underlying high-resolution 3D image ***ρ*** (in the end-expiration reference respiratory motion state), the soft-gated and translational-motion-corrected k-space **b** is obtained. That is,

(1) b=Eρ.

Here, $\rho \in C^{N\times 1}$ is arranged as a vector of voxel values, where *N* is the total number of voxels in the high-resolution 3D image, and $b\in C^{N_{c}\left(\sum_{i=1}^{N_{b}}K_{i}\right)\times 1}$ is a vector containing the k-space sample values for each of the *N*_*b*_ respiratory bins and *N*_*c*_ coils, where *K*_*i*_ is the number of k-space samples in the *i*th bin. We note that the total number of acquired k-space samples, *K*, is not equal to $\sum_{i=1}^{N_{b}}K_{i}$, since the soft-gating leads to the same sample being included with different weights in multiple bins. The encoding operator may be expressed as

(2) E=WDFCM,

where $M\in R^{N_{b}N\times N}$ applies 3D non-rigid motion warping to obtain images in each respiratory motion state from the reference-bin image, $C\in C^{N_{c}N_{b}N\times N_{b}N}$ contains *N*_*b*_ replicas of the coil-sensitivity maps, which multiply each image to form coil-weighted images, $F\in C^{N_{c}N_{b}N\times N_{c}N_{b}N}$ applies the 3D Fourier transform to each coil-weighted bin image, $D\in N^{N_{c}\left(\sum_{i=1}^{N_{b}}K_{i}\right)\times N_{c}N_{b}N}$ is the down-sampling operator, which selects only those k-space readouts that were acquired during the scan, removing both unacquired readouts from the center of the *k*_*y*_*k*_*z*_-plane and those readouts located in the outer region of the *k*_*y*_*k*_*z*_-plane which were not acquired since the scan was conducted at low resolution, and $W\in R^{N_{c}\left(\sum_{i=1}^{N_{b}}K_{i}\right)\times N_{c}\left(\sum_{i=1}^{N_{b}}K_{i}\right)}$ is a diagonal matrix containing the soft-gating weights between 0 and 1.

The zero-filled reconstruction of each respiratory bin image was calculated as

(3) $$\rho_{ZF}=C^{*}F^{-1}D^{T}W^{T}b,$$

where * denotes the conjugate transpose. This expression is equivalent to E*b except for the absence of M*, which could not be applied initially since the motion fields were estimated within the framework. Ultimately, M* was applied to ***ρ***_*ZF*_ during the data consistency step of the reconstruction, as described below.

#### 2.2.2 Splitting zero-filled images into patches

Due to memory constraints, the zero-filled images ***ρ***_*ZF*_ were split into overlapping patches in the readout (*x*) direction. Since the acquisition is fully sampled at high resolution in this direction, low-resolution and undersampling artifacts propagate in the *yz*-plane only. Following reconstruction, the high-resolution output patches were combined to form a high-resolution 3D whole-heart image.

#### 2.2.3 Non-rigid motion estimation

Each zero-filled bin image was passed through an iterative sensitivity encoding (SENSE) [84] reconstruction to produce a de-noised auxiliary image. These were then passed pairwise into a diffeomorphic motion estimation network [34] which has been utilized in previous MoCo-MoDL studies [28], [29] and incorporates a 3D U-Net structure followed by a scaling and squaring layer, ensuring the output motion fields are diffeomorphic [35]. A diagram depicting the exact architecture of the motion estimation network is included in Supplementary File: Section 2.

#### 2.2.4 Iterative MoDL reconstruction

The task of reconstructing the high-resolution whole-heart image from low-resolution and potentially undersampled k-space data can be formulated as the constrained optimization problem

(4) $$\arg\;\underset{\rho ,\omega }{\min}\left\{\frac{1}{2}{∥E\rho -b∥}_{2}^{2}+\frac{\mu }{2}{∥\omega -S\left(\omega \right)∥}_{2}^{2}\right\}\;\;\text{subject to}\;\;\rho =\omega .$$

Here, ***ω*** is an additional image-space variable, constrained to be equal to the high-resolution image ***ρ***, *S*(. ) represents the action of the super-resolving U-Net, which, when correctly trained, will super-resolve and denoise its input, and *μ* is a penalty weighting parameter that determines the relative importance of the first term, which ensures data consistency is maintained in the acquired lines of k-space, and the second, which forces the solution to lie within the subset of the *N*-dimensional hyperspace that contains high-resolution images. A diagram depicting the exact architecture of the SR U-Net is included in Supplementary File: Section 2.

In previous implementations of MoDL and MoCo-MoDL [28], [29], [31], an alternating iteration was employed to solve the equivalent equation, but this was found to result in unstable network training in the present application. Instead, an ADMM (alternating direction method of multipliers) [85] scheme, as suggested in the original MoDL paper [31], was employed in order to achieve greater stability. In Eq. (4), the objective has been split across two variables, ***ρ*** and ***ω***, which are constrained to be equal. By making this choice, the corresponding scaled Augmented Lagrangian [85] may be written as

(5) $$\begin{array}{ccc} L\left(\rho ,\omega ,a\right) & = & \frac{1}{2}{∥E\rho -b∥}_{2}^{2}\\ & & +\frac{\mu }{2}{∥\omega -S\left(\omega \right)∥}_{2}^{2}+\frac{\lambda }{2}{∥\rho -\omega +a∥}_{2}^{2}, \end{array}$$

where *λ* is the penalty parameter and **a** is the scaled dual variable. The ADMM scheme to minimize L yields a repeating sequence of the following three steps:

(6) $$\begin{array}{ccc} 1.\;\rho^{\left(j+1\right)} & = & \arg\;\underset{\rho }{\min}\left\{\frac{1}{2}{∥E\rho -b∥}_{2}^{2}\right)\\ & & +\left(\frac{\lambda }{2}{∥\rho -\omega^{\left(j\right)}+a^{\left(j\right)}∥}_{2}^{2}\right\} \end{array}$$

(7) $$\begin{array}{ccc} 2.\;\omega^{\left(j+1\right)} & = & \arg\;\underset{\omega }{\min}\left\{\frac{\mu }{2}{∥\omega -S\left(\omega \right)∥}_{2}^{2}\right)\\ & & +\left(\frac{\lambda }{2}{∥\rho^{\left(j+1\right)}-\omega +a^{\left(j\right)}∥}_{2}^{2}\right\} \end{array}$$

(8) $$3.\;a^{\left(j+1\right)}=a^{\left(j\right)}+\rho^{\left(j+1\right)}+\omega^{\left(j+1\right)},$$

as depicted in Fig. 1.

Step 3 can be applied directly, while Step 1 can be solved iteratively via conjugate gradient (CG) descent. The CG algorithm utilizes the fact that the first term of the objective in Eq. (6) is minimized when E*E***ρ*** = E*b, as illustrated in Fig. 1. Noting that E*b = M*ρ_*ZF*_, this reveals how genuine data consistency can be enforced when it is the zero-filled images ***ρ***_*ZF*_, rather than the k-space data **b**, that are input to the framework.

To implement Step 2, we first define ${\overset{˜}{\omega }}^{\left(j\right)}$ as

(9) $${\overset{˜}{\omega }}^{\left(j\right)}=\rho^{\left(j+1\right)}+a^{\left(j\right)}.$$

We now make the assumption that *S* is such that, for all images **t**,

(10) $$\arg\;\underset{d}{\min}\left\{{∥t-S\left(d\right)∥}_{2}^{2}\right\}=t,$$

where the optimization variable $d\in C^{N}$. That is, when an arbitrary image **t** is input to the SR U-Net, the output is the closest image that lies in T, the set of all output images;

(11) $$T=\left\{\omega :\omega =S\left(t\right),t\in C^{N}\right\}.$$

Thus, if an already-high-resolution image is input to S, S acts as an identity operator and returns the image unchanged. Since this assumption is built into the ADMM MoDL iteration, it is expected to be learnt during training.

Given Eq. (10), it can be shown that the solution to Eq. (7) is

(12) $$\omega^{\left(j+1\right)}=\left(\frac{\mu }{\mu +\lambda }\right)S\left({\overset{˜}{\omega }}^{\left(j\right)}\right)+\left(\frac{\lambda }{\mu +\lambda }\right){\overset{˜}{\omega }}^{\left(j\right)},$$

which provides a straightforward way to implement Step 2 of the ADMM iteration. A derivation of this result is included in Supplementary File: Section 3.

The iterative reconstruction thus proceeds by implementing Eqs. (6), (12), and (8) successively until a predetermined number of iterations has been reached.

### 2.3 Preparation of training data

**Fig. 2:**
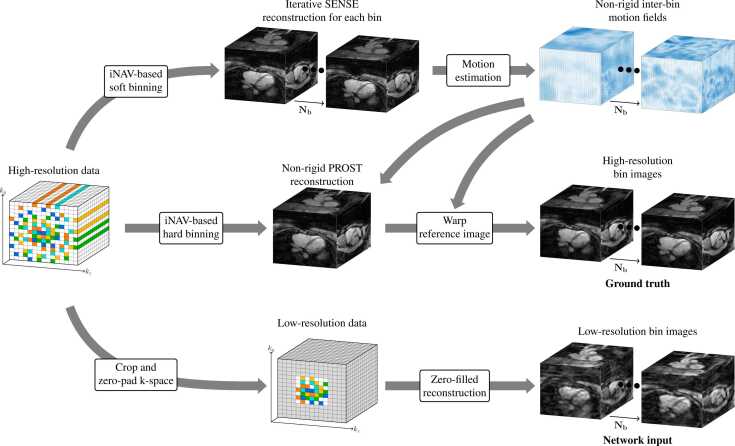
Generation of training data from isotropic-resolution data. Iterative SENSE reconstructions of each respiratory bin are used in conventional motion estimation, with the corresponding motion fields incorporated into an NR-PROST reconstruction, which is treated as the ground truth for training. Corresponding undersampled and low-resolution images are obtained via retrospective k-space down-sampling followed by a zero-filled reconstruction. *NR-PROST* non-rigid motion-corrected patch-based low-rank reconstruction method, *SENSE* sensitivity encoding

Training data were generated from undersampled isotropic-resolution scans, as depicted in Fig. 2. For each respiratory bin, a high-resolution 3D image and corresponding low-resolution zero-filled image were generated, with the low-resolution-reconstruction procedure designed so its output would match the images from the prospective low-resolution acquisitions.

#### 2.3.1 High-resolution images

Initially, the iNAV-based translational motion correction and soft-gated respiratory binning procedure, as described previously for prospective low-resolution acquisitions, was applied to the high-isotropic-resolution data. Additionally, the k-space was zero-padded in the *k*_*y*_- and *k*_*z*_-directions to the next multiple of 16, ensuring that the max-pooling and up-sampling layers of the deep neural networks did not need to handle any odd array sizes (this was also guaranteed in the *k*_*x*_-dimension by setting the patch thickness to a multiple of 16).

An iterative SENSE reconstruction was implemented for each respiratory bin to generate intermediate bin images, from which non-rigid respiratory motion fields were estimated using the software NiftyReg [86]. A non-rigid motion-corrected patch-based low-rank (NR-PROST) reconstruction [12], [13] incorporating the NiftyReg motion fields was implemented, resulting in a high-resolution image for the reference respiratory bin. The remaining bin-images were obtained by warping the reference-bin image with the NiftyReg motion fields, as shown in Fig. 2. The resultant high-resolution multi-bin 3D images formed the ground truth for training.

Since the degree of undersampling in the isotropic-resolution acquisitions was limited to 3- or 4-fold (as detailed in Table 1), the NR-PROST reconstruction approach yielded high-quality isotropic-resolution images, which were thus suitable to serve as the reference images during the training of Super-MoCo-MoDL for further-undersampled data.

**Table 1:** Details of the isotropic-resolution training and retrospective test datasets

		Isotropic	VD-CASPR	No. Patients
Condition	Sequence	Resolution	Undersampling	(Train/Test)
CHD	Single Contrast	1.5 mm	3	6 / 1
CHD	Single Contrast	1.5 mm	4	28 / 4
CHD	MTC-BOOST	1.4 mm	3	3 / 1
CHD	MTC-BOOST	1.5 mm	3	92 / 8
TA	i*T*_2_-prep-BOOST	1.3 mm	4	23 / 0
TA	i*T*_2_-prep-BOOST	1.4 mm	3	2 / 1
TA	i*T*_2_-prep-BOOST	1.6 mm	3	2 / 0
				**156 / 15**

Data are numbers of patients acquired with each combination of sequence parameters, allocated to training and test sets. *BOOST* bright-blood and black-blood phase sensitive, *CHD* congenital heart disease, *iT2-prep* interleaved T2-preparation, *MTC* magnetization transfer contrast, *TA* thoracic aortopathy, *VD-CASPR* variable density Cartesian acquisition with spiral profile order

#### 2.3.2 Low-resolution images

The corresponding low-resolution zero-filled images (with the same array sizes) were formed via retrospective down-sampling. Diagrams depicting the retrospective down-sampling process in detail for both the 2 × 2 and 4 × 4 SR schemes are included in Supplementary File: Section 4. We have previously demonstrated, for the case of a retrospectively increased undersampling factor, that retaining a subset of the acquired k-space readouts, rather than using k-space values synthesised from the high-resolution image, achieves a closer match between training and prospective data and leads to improved image quality [29]. As such, k-space sampling masks (in the *k*_*y*_*k*_*z*_-plane) from prospective low-resolution scans were segmented into 20 concentric elliptic annuli and the average sampling density in each annulus was calculated. The 20-annuli segmentation was then overlaid on the high-isotropic-resolution k-space data, with the SR factor determining the scale of the annuli. All k-space lines located outside the outermost annulus were discarded, while within each annulus acquired lines were randomly selected to be retained until the measured sampling density was matched. Finally, a zero-filled reconstruction of the remaining k-space data was implemented for each respiratory bin to provide the low-resolution zero-filled images for network input during training.

### 2.4 Data acquisition

The study was approved by the National Research Ethics Service (15/NS/0030, 18/SC/0441) and written informed consent was obtained from each participant according to institutional guidelines.

#### 2.4.1 Training data

171 patients with either congenital heart disease (CHD) (143 patients) or thoracic aortopathy (28 patients) were scanned on a 1.5-T MRI scanner (MAGNETOM Aera, Siemens Healthcare, Erlangen, Germany) using one of the three sequences described in Section 2.1 at isotropic resolution between 1.3 and 1.6 mm with 3- or 4-fold VD-CASPR undersampling. These data were randomly sorted into a training set (156 patients) and a retrospective test set (15 patients). Further details on the composition of these data sets are provided in Table 1.

#### 2.4.2 Prospective data

Prospective data were acquired using two SR schemes as follows: approximately full VD-CASPR sampling with 4 × 4 SR in the *k*_*y*_*k*_*z*_-plane, and 4.5-fold VD-CASPR undersampling with 2 × 2 SR in the *k*_*y*_*k*_*z*_-plane, as shown in Table 2. Both schemes led to an overall undersampling factor of ∼ 18.

**Table 2:** Details of the prospective low-resolution datasets

Condition	No. Patients	Anisotropic Acquired Resolution	Isotropic Target Resolution	Av. VD-CASPR Undersampling	Av. SR Factor	Av. Overall Undersampling
CHD	5	1.5 mm × 6 mm × 6 mm	1.5 mm	1.15	16.33	18.8±0.3
Suspected CAD	35	0.9 mm × 1.8 mm × 1.8 mm	0.9 mm	4.58	4.04	18.5±0.2

Average SR factors are slightly larger than the integers expected due to zero-padding to a multiple of 16. Note that VD-CASPR and overall undersampling values apply within an elliptical shutter; values would increase by a factor of 4∕π if expressed relative to the rectangular field of view. *CAD* coronary artery disease, *CHD* congenital heart disease, *SR* super resolution, *VD-CASPR* variable density Cartesian acquisition with spiral profile order

For the 4 × 4 SR scheme, each of the 39 CHD patients scanned with the single-contrast sequence (see Table 1) were also scanned with the same sequence at anisotropic 1.5 mm × 6 mm × 6 mm resolution. Of these, only the five patients whose isotropic-resolution scans were not included in the training set had their prospective low-resolution scans (Table 2) reconstructed with Super-MoCo-MoDL and analyzed.

The cohort scanned with the 2 × 2 SR scheme comprised 35 patients with suspected coronary artery disease (CAD). These patients first underwent a computed tomography coronary angiography (CTCA) scan as part of standard of care for investigation of CAD. All CTCA scans were performed using a dual-source CT system (SOMATOM Force, Siemens Healthcare, Forchheim, Germany) with ECG synchronisation via a three-lead vector ECG to enable cardiac gating. Provided no contraindications, patients received 800 *μ*g of sublingual GTN (glyceryl trinitrate) to promote coronary vasodilation prior to image acquisition. Where appropriate, intravenous (IV) metoprolol (Betaloc, AstraZeneca, London, United Kingdom) was administered in 2.5 mg increments to achieve a target heart rate below 65 beats per minute (bpm). Image acquisition utilized a prospectively ECG-triggered axial protocol during an inspiratory breath hold. The ECG pulsing window and associated padding parameters were determined automatically by the scanner software. A triphasic contrast injection protocol was employed at a flow rate of 6 mL/second. The standard regimen included 70 mL of undiluted iodinated contrast agent (Iohexol 370 mg/mL; Omnipaque, GE Healthcare, London, United Kingdom), followed by 75 mL of a contrast and saline mixture (30%:70%, respectively), and concluded with a 25 mL saline flush. Bolus tracking was used to initiate scanning once the attenuation in the proximal descending aorta reached a threshold of 110 Hounsfield units. Image reconstruction was carried out using the Advanced Modelled Iterative Reconstruction algorithm (ADMIRE, Siemens Healthineers, Forchheim, Germany) to achieve a final spatial resolution of 0.6 mm isotropic.

These suspected-CAD patients were prepared in a similar manner for MRI scanning, with sublingual GTN and IV metoprolol where appropriate. Imaging was performed in diastole where a target heart rate of < 70 beats per minute was achieved, and in systole otherwise. These scans were conducted on a 1.5-T MRI scanner (MAGNETOM Sola, Siemens Healthcare, Erlangen, Germany) using the single-contrast sequence at anisotropic 0.9 mm × 1.8 mm × 1.8 mm resolution (Table 2). For comparison, these patients were also scanned at isotropic 0.9 mm resolution with 4.5-fold VD-CASPR undersampling.

### 2.5 Pre-training the super-resolving U-Net

Before end-to-end training of the entire Super-MoCo-MoDL framework, the super-resolving U-Net was pre-trained on 2315 scans from the NYU fastMRI dataset [87], including 1343 multi-coil brain scans and 972 single-coil knee scans. A diagram depicting the training scheme is included in Supplementary File: Section 2. For the multi-coil data, a single coil was selected at random and only data from that channel was utilized.

The pre-training required pairs of high- and low-resolution 3D images from each scan. Initially, the k-space data from each scan was zero-padded and/or cropped to form a 32 × 320 × 320 array. The high-resolution image was then obtained by applying the inverse Fourier transform directly to this k-space. To obtain low-resolution images with the same degree of resolution reduction and undersampling artifacts expected in the Super-MoCo-MoDL reconstruction, the previously described retrospective down-sampling scheme was applied, followed by an inverse Fourier transform.

A perceptual loss function [88], which more strongly penalises overall perceived image quality and sharpness than pixel-wise losses, was evaluated between the high-resolution ground truth image (***ρ***_hr_) and the super-resolved network output (***ρ***_out_). The perceptual loss was calculated as

(13) $$L_{\mathrm{perc}}=\sum_{\nu =1}^{5}\psi_{c_{\nu },d_{\nu }}L_{\mathrm{feat},c_{\nu },d_{\nu }},$$

a weighted sum of losses calculated on five individual feature maps of the VGG-19 network [89] after each of the images was given as input. Specifically, *L*_feat,*c*,*d*_ is the mean squared error on the feature map in the *c*th block of the network after the *d*th convolution, given as

(14) $$\begin{array}{ccc} L_{\mathrm{feat},c,d} & = & \frac{1}{N_{x}W_{c,d}H_{c,d}}\\ & & \times \overset{N_{x}}{\underset{x=1}{\sum }}\overset{W_{c,d}}{\underset{\alpha =1}{\sum }}\overset{H_{c,d}}{\underset{\beta =1}{\sum }}{\left[{\left[\phi_{c,d}\left(\rho_{\mathrm{hr},x}\right)\right]}_{\alpha ,\beta }-{\left[\phi_{c,d}\left(\rho_{\mathrm{out},x}\right)\right]}_{\alpha ,\beta }\right]}^{2}, \end{array}$$

where *ϕ*_*c*,*d*_ is the feature map and *W*_*c*,*d*_ and *H*_*c*,*d*_ are its dimensions. *N*_*x*_ is the size of the fully sampled readout (*x*) direction. Since VGG-19 has a 2D architecture, slices in this direction corresponded to the input batch dimension.

The feature maps and corresponding weights utilized were: *ϕ*_1,1_, *ψ*_1,1_ = 3.0; *ϕ*_2,2_, *ψ*_2,2_ = 0.04; *ϕ*_3,3_, *ψ*_3,3_ = 0.016; *ϕ*_4,3_, *ψ*_4,3_ = 0.01; *ϕ*_5,4_, *ψ*_5,4_ = 14.0; the same as those applied in a previous study applying the MoCo-MoDL framework to 2D cine reconstruction [90]. In that study, weights were chosen via a manual parameter search to ensure each weighted feature-map loss contributed approximately equally to the combined perceptual loss, a property that was also observed for the present application.

The total pre-training loss was calculated as

(15) $$L_{\mathrm{pretrain}}=\gamma_{1}L_{\mathrm{perc}}+\gamma_{2}L_{\mathrm{SRreg}},$$

where *L*_SRreg_ is a regularization loss on the super-resolving U-Net, calculated as the *l*^2^-norm of the convolution kernel parameters.

Pre-training was implemented on a 16-GB GPU (graphics processing unit) for 400 epochs, utilizing the Adam optimizer[91] with a learning rate of 1 × 10^−5^. The penalty weights multiplying the losses were set to *γ*_1_ = 100 and *γ*_2_ = 1. Training was conducted separately for the 4 × 4 and 2 × 2 SR schemes.

### 2.6 End-to-end training

The entire framework was trained end-to-end using the 156-patient training dataset with the parameters of the super-resolving U-Net initialized to the result of the pre-training procedure and then fine-tuned. The training loss was calculated as

(16) $$L=\eta_{1}L_{\mathrm{perc}}+\eta_{2}L_{\mathrm{reg}}+\eta_{3}L_{\mathrm{mot}},$$

where the perceptual loss *L*_perc_ was identical to that already described for pre-training and was calculated between the motion-informed MoDL output and the high-resolution reference image, the regularization loss *L*_reg_ was here calculated as the *l*^2^-norm of the convolution kernel parameters of both networks, and the motion loss *L*_mot_ was calculated as the Charbonnier loss between the end-expiration high-resolution reference image and the reference images from other respiratory bins warped to the reference bin by the estimated motion fields. In this sense, the motion estimation component of the framework was self-supervised; no ground-truth non-rigid motion fields were required for end-to-end training.

Despite padding the k-space arrays to a multiple of 16 in each of the *k*_*y*_ and *k*_*z*_ dimensions, without further adjustment 33 unique array sizes and 23 unique *k*_*y*_-*k*_*z*_ slice sizes would exist in the training set. Since patching was applied in the *x* direction, the number of unique *k*_*y*_-*k*_*z*_ slices determined the number of unique patch sizes, and memory constraints arose when a large number of unique patch sizes, or any too-large patches, existed in the training set. Consequently, additional k-space cropping and/or padding was applied to 35 of the 156 patients in the training set, reducing the number of unique slice sizes from 23 to 7, as listed in Table 3. Tables detailing all array sizes before and after this process are included in Supplementary File: Section 5.

**Table 3:** Number of *k*_*y*_-*k*_*z*_ arrays with specific slice sizes in the training and retrospective test sets after k-space padding and cropping

***k***_***y***_-***k***_***z***_	Training Set			Retrospective Test Set		
Slice Size	SC	MTC	i***T***_**2**_-prep	SC	MTC	i***T***_**2**_-prep
272 × 112	11	–	1	1	–	–
272 × 128	6	3	–	1	1	–
288 × 96	1	3	–	–	–	–
288 × 112	7	6	1	3	–	–
288 × 128	7	29	–	–	1	–
304 × 112	1	15	2	–	–	–
304 × 128	1	39	23	–	5	–
304 × 144	–	–	–	–	2	–
320 × 128	–	–	–	–	–	1

Data are numbers of arrays of each slice size appearing in the training and retrospective test sets. *SC* single contrast, *MTC* magnetization transfer contrast, *iT2-prep* interleaved T2-preparation

For each of the 4 × 4 and 2 × 2 SR schemes, the training was implemented on two 16-GB GPUs for 1000 epochs, utilizing the Adam optimizer [91] with an initial learning rate of 1 × 10^−4^, which was reduced by a factor of 2 after 500 epochs. The penalty weights multiplying the losses were set to *η*_1_ = 10, *η*_2_ = 1 and *η*_3_ = 100. These parameters were determined following a limited qualitative optimization, starting from values used in a previous MoCo-MoDL study [29].

The *x*-direction patch size was set to 32, and each epoch, for every training-set patient, a different 32-voxel-thick 3D patch from the central 50% of the *x*-axis was randomly selected. Motion fields were estimated using patches of the same size for all patients, achieved by padding (when necessary) to 32 × 304 × 128-sized patches then down-sampling by a factor of 2 in all dimensions. When padding was applied to a patch before input to the motion-estimation network, cropping was applied to the output non-rigid motion fields to ensure their dimensionality was consistent with the different patch sizes, which were used throughout the ADMM-based MoDL reconstruction.

Other parameters applied during training and inference, selected following the limited hyper-parameter search, were: *λ* = 1.5, *μ* = 10, no. of CG iterations during first ADMM step = 5, no. of ADMM iterations = 4 (stopping after Step 1 in the final iteration), no. of iterative SENSE iterations for auxiliary patch de-noising prior to motion estimation = 10.

### 2.7 Reconstruction

The trained Super-MoCo-MoDL frameworks were applied patch-wise to reconstruct all retrospective and prospective low-resolution datasets. To avoid edge effects from patch recombination, a five-voxel edge layer of each patch was discarded, leaving a four-voxel overlap between adjacent patches.

For comparison, NR-PROST was applied to the low-resolution data in two ways. In the first, NR-PROST was directly applied to the small-array low-resolution data, and the output images were interpolated using bicubic interpolation to the high-resolution array size. In the second, the k-space data were zero-padded to the high-resolution array size and NR-PROST was applied to these larger arrays.

For comparison, NR-PROST was also applied to all high-resolution yet undersampled datasets, providing a high-resolution reference image.

### 2.8 Image analysis

The quantitative error metrics mean squared error (MSE) and structural similarity (SSIM) were calculated relative to the reference high-resolution NR-PROST images for all 15 subjects in the retrospective test set, using a manually selected 3D region of interest (ROI) around the heart. For each subject, the reference image was normalized to have a maximum absolute pixel value of 1 within the ROI and all reconstructions from low-resolution data were scaled separately to optimize the metrics. The statistical significance of the differences between mean error-metric values was calculated using paired-samples t-tests, with the significance criterion set as *p*<0.0033 following Bonferroni correction for 15 pair-wise comparisons. These pairs were formed from six separate reconstructions of each image (three reconstruction methods and two SR schemes).

For prospective reconstructions, the high-resolution reference images were obtained from separate successive scans, and so direct pixel-wise comparisons were not applicable. Instead, qualitative scoring of overall image quality, alongside wall sharpness and robustness to artifacts for ten vascular structures, was performed by a cardiologist blinded to reconstruction type (S.J.L., 3 years experience) on a 5-point Likert Scale defined as: 1 - non-diagnostic; 2 - poor image quality (poor endocardial/vessel wall definitions and/or significant noise/artifact); 3 - adequate image quality (overall sufficient image quality, but one or more structures may be less well-defined or be subject to noise/artifact); 4 - good image quality (structures generally well-defined but may be some noise/artifact); 5 - excellent image quality (sharp definitions of all structures without any significant noise/artifact). Example images that were awarded various overall image quality scores are included in Supplementary File: Section 6. A best-to-worst overall quality ranking of the three reconstructions and the reference scan for each patient was also performed, with the cardiologist again blinded to reconstruction type.

For the 35 suspected-CAD-patient scans (Table 2), the statistical significance of the quality scores was assessed using a Wilcoxon signed rank test, with the significance criterion set to *p* < 0.0083, with a Bonferroni correction made for 6 pair-wise comparisons. For the 5 CHD patients (Table 2), statistical significance of the quality scores was not assessed, since with only 5 scores the Wilcoxon signed rank test has no statistical power (*p*>0.05 even when one set of scores are all greater than, or all less than, another set).

Quantitative evaluation of vessel sharpness in the suspected-CAD-patient prospective images was performed using the software “Soap-Bubble”[92]. Images were reformatted along the left anterior descending artery (LAD) and right coronary artery (RCA), and the average sharpness along the first 4 cm of each was calculated. A sharpness score of 0% indicated the absence of an edge, while a score of 100% implied an immediate transition to the maximum intensity seen in the vessel. A paired-samples t-test was again used to determine the statistical significance of the difference in mean value. The significance criterion was again set as *p*<0.0083 due to the inclusion of 6 pair-wise comparisons.

## 3 Results

The average acquisition time of the 1.5 mm × 6 mm × 6 mm anisotropic CHD-patient scans was 0.8 ± 0.2 min, compared to the average for the 1.5-mm isotropic-resolution scans of the same patients, which was 3.3 ± 0.4 min.

The average acquisition time of the 0.9 mm × 1.8 mm × 1.8 mm anisotropic suspected-CAD-patient scans was 2.1 ± 0.3 min, a ∼ 4-fold speed-up relative to the average for the 0.9-mm isotropic-resolution scans of 8.3 ± 1.0 min.

The SR U-Net pre-training (400 epochs) took ∼ 189 and ∼ 176 h for the 4 × 4 and 2 × 2 SR schemes, respectively, and the subsequent end-to-end training (1000 epochs) took ∼ 207 and ∼ 209 h, respectively. Once the framework was trained, reconstruction with the Super-MoCo-MoDL framework took 54 ± 16 s for each CHD patient and 126 ± 18 s for the suspected-CAD patients. The difference in these reconstruction times is due to the larger k-space array sizes of the suspected-CAD patients, which are reconstructed to 0.9-mm isotropic resolution, as opposed to the 1.5-mm isotropic resolution of the CHD patients.

### 3.1 Retrospective test results

**Fig. 3:**
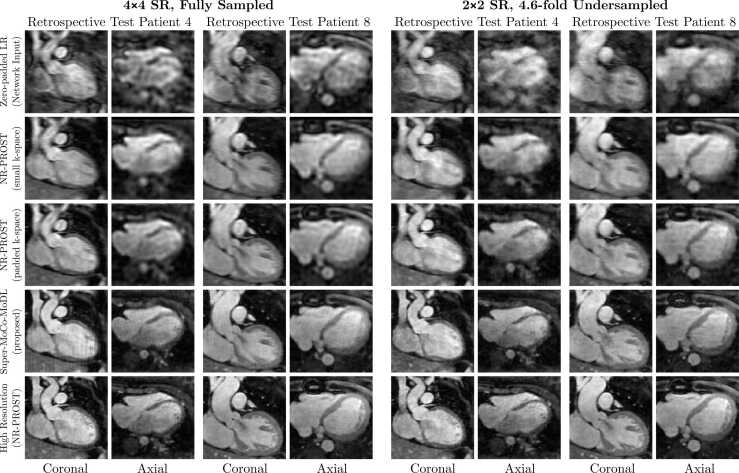
Coronal and axial slices of the NR-PROST reconstruction of high-resolution data (bottom row) and the same slices from reconstructions of low-resolution data obtained by retrospectively undersampling the high-resolution data using the 4 × 4-SR scheme (left) and the 2 × 2 scheme (right), for two example patients from the retrospective test set. The reconstruction methods for low-resolution data are: zero-filled reconstruction of reference respiratory bin (1st row), NR-PROST applied to the small-array low-resolution k-space array followed by bicubic interpolation to the high-resolution image size (2nd row), NR-PROST applied to zero-padded low-resolution k-space (3rd row) and the proposed Super-MoCo-MoDL approach (4th row). Each image is individually normalized. *LR* low resolution, *NR-PROST* non-rigid motion-corrected patch-based low-rank reconstruction method, *SR* super resolution

Reconstructions for two example patients allocated to the retrospective test set are presented in Fig. 3, for both the 4 × 4 and 2 × 2 SR schemes. Visually, the Super-MoCo-MoDL reconstructions are seen to be sharper than either NR-PROST reconstruction of the low-resolution datasets, although the image quality of the high-resolution NR-PROST reconstruction is still superior. A weak vertical-lining artifact can be seen in the coronal plane of the 4 × 4-SR reconstructions and is also visible in the axial plane, where it manifests as a pixellation-like pattern. This artifact is less evident in the 2 × 2-SR reconstructions, which also appear sharper.

**Fig. 4:**
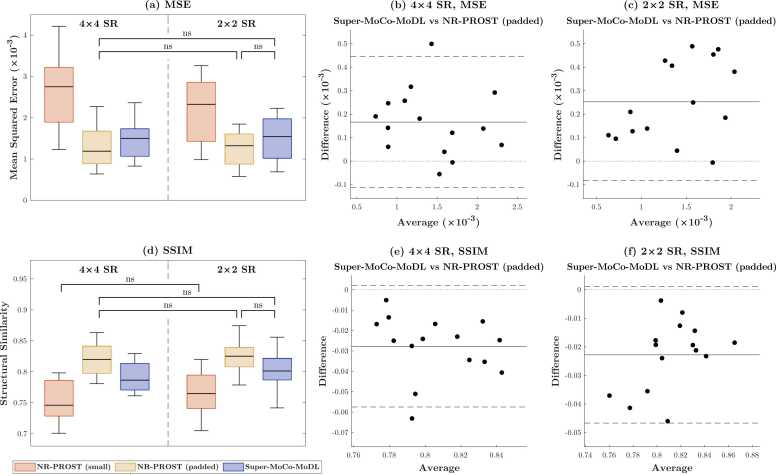
Quantitative error metrics MSE (top) and SSIM (bottom) calculated with the 15-patient retrospective test set relative to NR-PROST reconstructions of high-resolution data. Boxplots (left) depict the values achieved using NR-PROST on small-array low-resolution k-space followed by bicubic interpolation, NR-PROST on a zero-padded k-space, and the proposed Super-MoCo-MoDL method, for both the 4 × 4 and 2 × 2 SR schemes. ‘ns’ denotes a non-significant difference, all other pairwise comparisons exhibited a statistically-significant difference (*p* < 0.0033 following Bonferroni correction for 15 comparisons). Bland-Altman plots (right) compare the values from Super-MoCo-MoDL and NR-PROST on zero-padded k-space pairwise, for both SR schemes. *MSE* mean squared error, *NR-PROST* non-rigid motion-corrected patch-based low-rank reconstruction method, *SR* super resolution, *SSIM* structural similarity

Results of the quantitative analysis of these retrospective reconstructions are presented in Fig. 4. The results indicate that both Super-MoCo-MoDL and NR-PROST applied to zero-padded k-space outperform the interpolated NR-PROST reconstruction as applied to a small unpadded k-space, with a statistically significant decrease in MSE and increase in SSIM observed for both the 4 × 4 and 2 × 2 SR schemes. For the 4 × 4 scheme, the mean ± standard deviation MSE and SSIM scores for the Super-MoCo-MoDL reconstructions ([1.52 ± 0.50] × 10^−3^ and 0.79 ± 0.02, respectively) are worse than those of padded NR-PROST ([1.35 ± 0.52] × 10^−3^ and 0.82 ± 0.03, respectively), despite the apparent increase in sharpness. For the 2 × 2 scheme, the difference is not statistically significant in either metric (*p *= 0.11 for MSE, *p *= 0.34 for SSIM). Comparing the Super-MoCo-MoDL reconstructions using the two SR schemes, the 2 × 2 scheme outperforms the 4 × 4 scheme with statistically significant differences recorded in both metrics (MSE: *p* = 3.3 × 10^−5^, SSIM: *p* = 2.9 × 10^−6^). No significant difference is observed between the SR schemes when considering the padded NR-PROST reconstructions (MSE: *p* = 0.16, SSIM: *p* = 0.56). Bland-Altman plots showcasing additional pairwise comparisons are included in Supplementary File: Section 7.

### 3.2 Prospective results

**Fig. 5:**
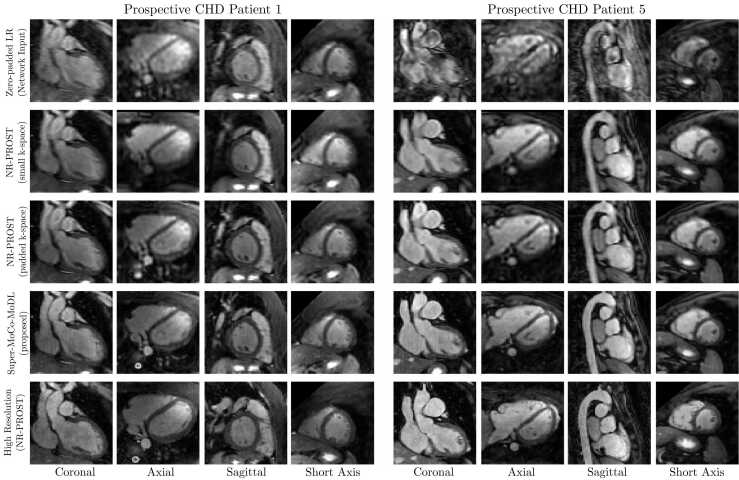
Coronal, axial, sagittal, and oblique short-axis slices of reconstructions of two prospective low-resolution scans of CHD patients acquired with the 4 × 4 SR scheme. The reconstruction methods for low-resolution data are: zero-filled reconstruction of reference respiratory bin (1st row), NR-PROST applied to the small-array low-resolution k-space array followed by bicubic interpolation to the high-resolution image size (2nd row), NR-PROST applied to zero-padded low-resolution k-space (3rd row) and the proposed Super-MoCo-MoDL approach (4th row). For comparison, NR-PROST reconstructions of high-resolution acquisitions of the same patients are included in the bottom row. Each image is individually normalized. *CHD* congenital heart disease, *LR* low resolution, *NR-PROST* non-rigid motion-corrected patch-based low-rank reconstruction method, *SR* super resolution

Reconstructions of prospective low-resolution datasets for two CHD patients (acquired with the 4 × 4 SR scheme) are presented in Fig. 5. Here, a view in each of the three orthogonal imaging planes is shown alongside an oblique short-axis view which can be readily generated from the isotropic-resolution images. As was the case for retrospective reconstructions using the 4 × 4 SR scheme, vertical-line artifacts are observed in both the coronal and sagittal planes of the Super-MoCo-MoDL reconstructions. A clear improvement in image sharpness is evident in the Super-MoCo-MoDL images in comparison to the other reconstructions of the same low-resolution data, with the visual quality approaching that of the high-resolution scans.

**Fig. 6:**
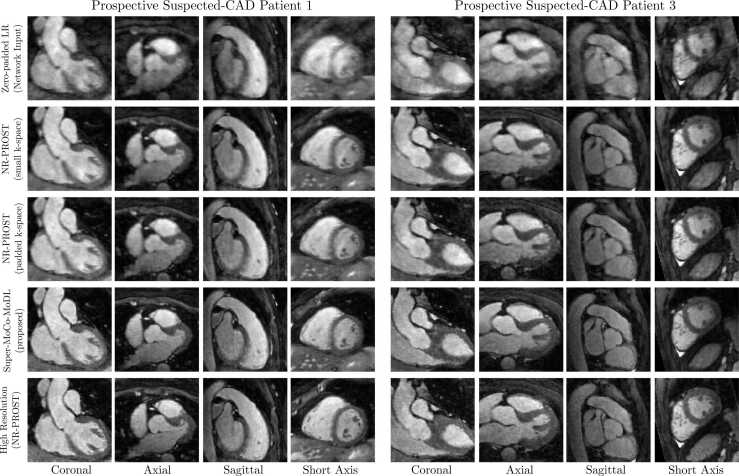
Coronal, axial, sagittal, and oblique short-axis slices of reconstructions of two prospective low-resolution scans of suspected-CAD patients acquired with the 2 × 2 SR scheme. The reconstruction methods for low-resolution data are: zero-filled reconstruction of reference respiratory bin (1st row), NR-PROST applied to the small-array low-resolution k-space followed by bicubic interpolation to the high-resolution image size (2nd row), NR-PROST applied to zero-padded low-resolution k-space (3rd row) and the proposed Super-MoCo-MoDL approach (4th row). For comparison, NR-PROST reconstructions of high-resolution acquisitions of the same patients are included in the bottom row. Each image is individually normalized. *CAD* coronary artery disease, *LR* low resolution, *NR-PROST* non-rigid motion-corrected patch-based low-rank reconstruction method, *SR* super resolution

Reconstructions of prospective low-resolution datasets for two suspected-CAD patients (acquired with the 2 × 2 SR scheme) are presented in Fig. 6. Again, slices in all three orthogonal planes and a short-axis view are included. No vertical-line artifacts, as seen in the 4 × 4-SR retrospective reconstructions, are observed in the Super-MoCo-MoDL images. Here, the overall image quality and sharpness achieved by Super-MoCo-MoDL is excellent, and comparable or superior to the prospective high-resolution NR-PROST images.

**Fig. 7:**
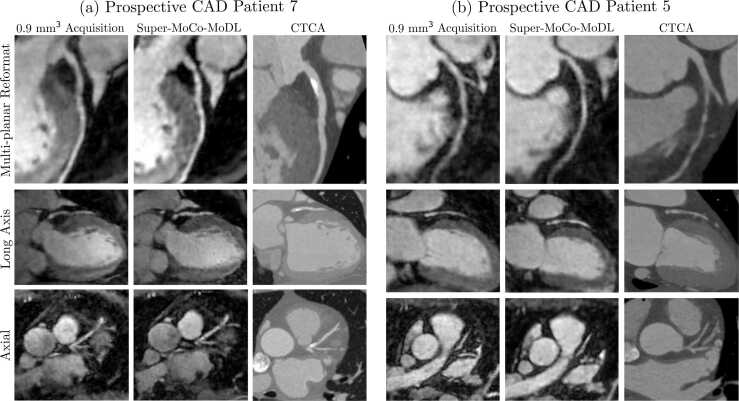
Comparison of NR-PROST reconstructions of high-resolution CMR acquisitions, the proposed Super-MoCo-MoDL approach as applied to prospective low-resolution CMR, and CTCA, in patients with CAD in the LAD. Rows depict: multi-planar reformats of the LAD (1st row), long-axis views of the proximal and mid LAD (2nd row) and axial views of the proximal and mid LAD (3rd row). (a) For CAD patient 7, white arrows indicate a large calcified plaque in the proximal vessel. (b) CAD patient 5 demonstrates non-calcified plaque in the proximal LAD, as indicated by white arrows. In (b), while still visible, the stenosis is less clearly visualized with Super-MoCo-MoDL than the isotropic acquisition. *CAD* coronary artery disease, *CMR* cardiac magnetic resonance, *CTCA* computed tomography coronary angiography, *LAD* left anterior descending artery, *NR-PROST* non-rigid motion-corrected patch-based low-rank reconstruction method

**Fig. 8:**
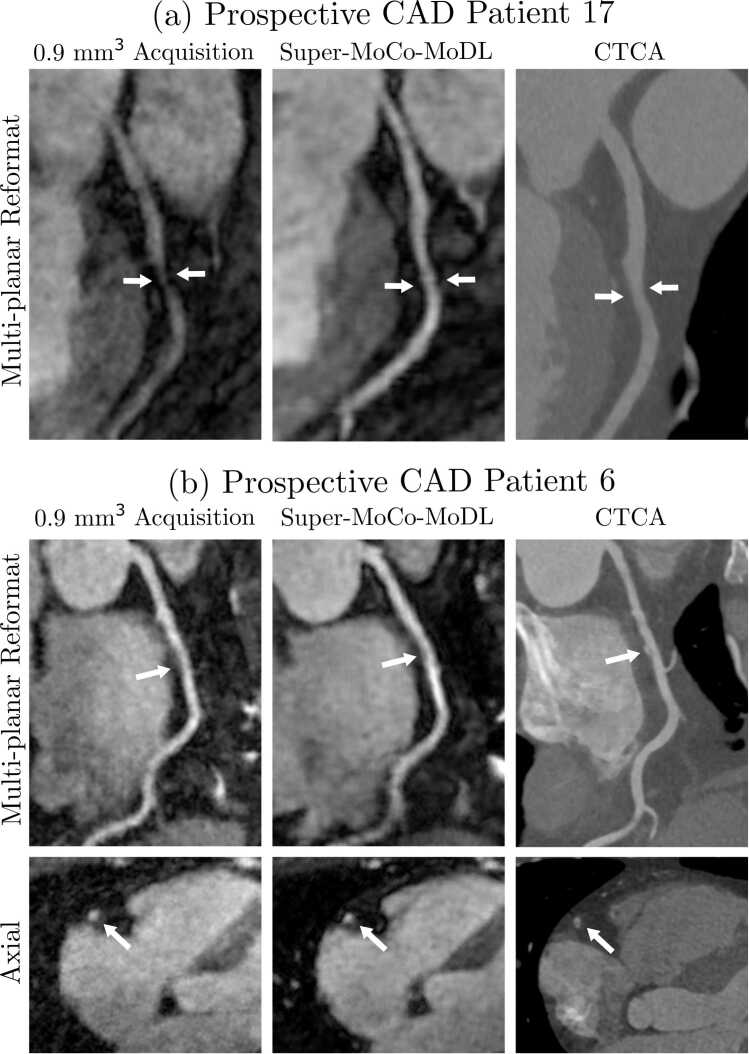
Comparison of NR-PROST reconstructions of high-resolution CMR acquisitions, the proposed Super-MoCo-MoDL approach as applied to prospective low-resolution CMR, and CTCA. (a) Multi-planar reformats of the LAD demonstrating CAD causing stenosis in the mid vessel (white arrows). The lesion appears more severe on the 0.9-mm isotropic CMRA compared to both the Super-MoCo-MoDL CMRA and the CTCA. Vessel wall contrast and delineation are improved with the Super-MoCo-MoDL reconstruction. (b) Multi-planar reformats (top) and axial views (bottom) of the RCA in a patient with mixed plaque in the mid vessel. White arrows indicate the location of the partially calcified plaque. *CAD* coronary artery disease, *CMR* cardiac magnetic resonance, *CMRA* coronary magnetic resonance angiography, *CTCA* computed tomography coronary angiography, *LAD* left anterior descending artery, *NR-PROST* non-rigid motion-corrected patch-based low-rank reconstruction method, *RCA* right coronary artery

Figures 7 and 8 showcase comparisons between high-resolution 0.9-mm isotropic CMR, Super-MoCo-MoDL reconstructions and CTCA, in patients with CAD. In all cases, the significant lesions seen on the CTCA were detected on both the high-resolution image and Super-MoCo-MoDL reconstruction despite different plaque morphologies.

**Fig. 9:**
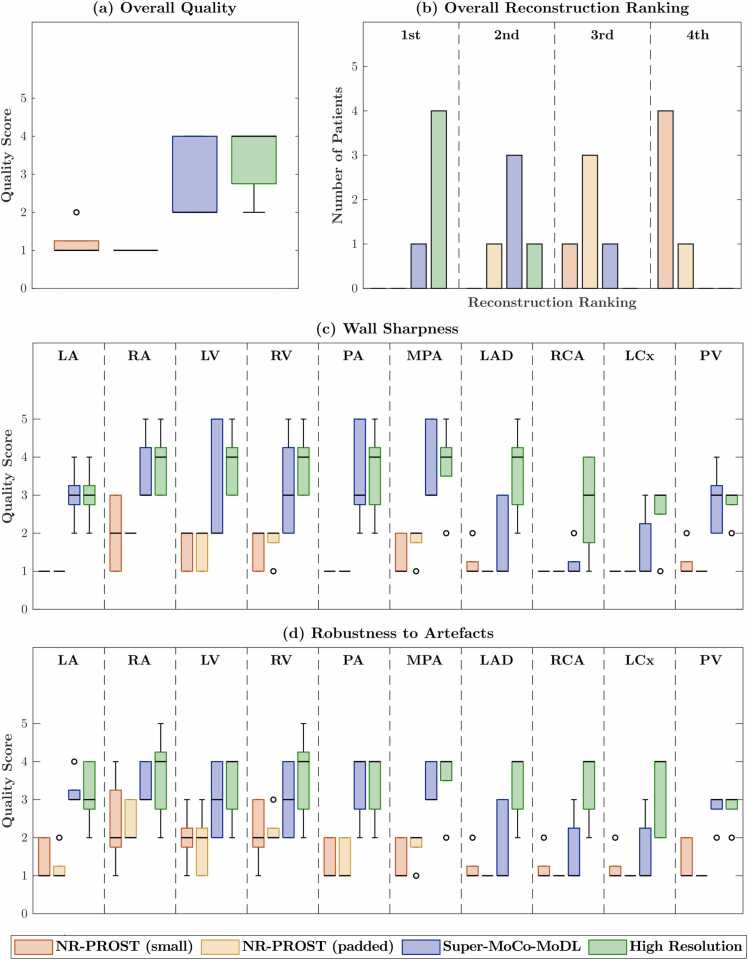
(a) Boxplots of overall image quality scores, (b) overall image rankings (1st - best; 4th - worst), and boxplots of (c) wall sharpness scores and (d) robustness-to-artifact scores, as assessed for the 5 CHD patients acquired with the 4 × 4 SR scheme. Scoring was performed for three reconstructions of prospective low-resolution data (small-k-space NR-PROST followed by bicubic interpolation, padded NR-PROST and the proposed Super-MoCo-MoDL approach) and for an NR-PROST reconstruction of the high-resolution acquisition. *CHD* congenital heart disease, *LA* left atrium, *LAD* left anterior descending artery, *LCx* left circumflex, *LV* left ventricle, *MPA* main pulmonary artery, *NR-PROST* non-rigid motion-corrected patch-based low-rank reconstruction method, *PA* proximal aorta, *PV* pulmonary veins, *RA* right atrium, *RCA* right coronary artery, *RV* right ventricle, *SR* super resolution

**Fig. 10:**
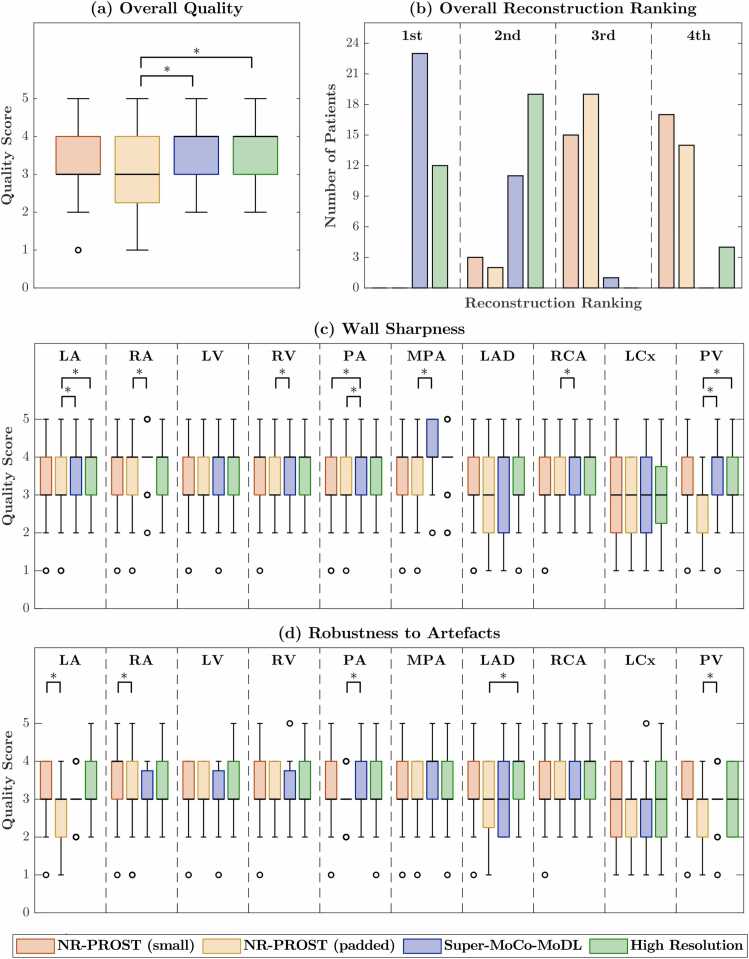
(a) Boxplots of overall image quality scores, (b) overall image rankings (1st - best; 4th - worst), and boxplots of (c) wall sharpness scores and (d) robustness-to-artifact scores, as assessed for the 35 suspected-CAD patients acquired with the 2 × 2 SR scheme. Scoring was performed for three reconstructions of prospective low-resolution data (small-k-space NR-PROST followed by bicubic interpolation, padded NR-PROST and the proposed Super-MoCo-MoDL approach) and for an NR-PROST reconstruction of the high-resolution acquisition. * denotes a statistically significant difference. *CAD* coronary artery disease, *LA* left atrium, *LAD* left anterior descending artery, *LCx* left circumflex, *LV* left ventricle, *MPA* main pulmonary artery, *NR-PROST* non-rigid motion-corrected patch-based low-rank reconstruction method, *PA* proximal aorta, *PV* pulmonary veins, *RA* right atrium, *RCA* right coronary artery, *RV* right ventricle, *SR* super resolution

Overall visual image quality scores, and qualitative vascular-structure-specific wall sharpness and robustness-to-artifact scores are presented for the 5 prospective CHD patients (4 × 4 SR) and 35 prospective suspected-CAD patients (2 × 2) in Figs. 9 and 10, respectively. While statistical significance could not be assessed for the 5 CHD patients, a consistent trend of higher scores from the Super-MoCo-MoDL reconstruction relative to the NR-PROST reconstructions of low-resolution data is observed. The scores for the suspected-CAD patients (Fig. 10) demonstrate similar values across the reconstruction methods, with few statistically significant differences observed. Figs. 9 and 10 also include the results of the overall image quality rankings. These results clearly demonstrate that, for both SR schemes, the Super-MoCo-MoDL reconstruction and high-resolution reference image tend to exhibit the best and second-best quality for a given patient, while the two NR-PROST reconstructions tend to rank third and fourth.

**Fig. 11:**
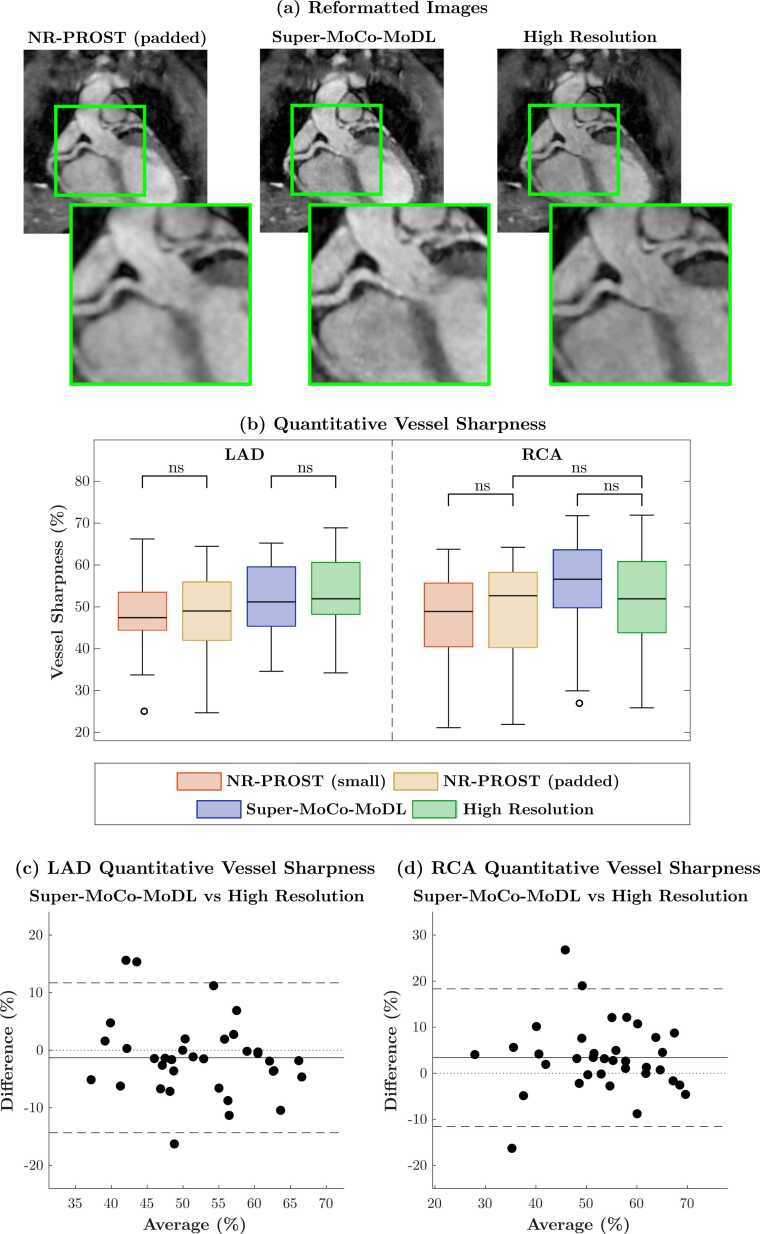
(a) Reformatted images for an example suspected-CAD patient (2 × 2 SR) depicting the LAD and RCA. (b) Boxplots of the average quantitative vessel sharpness score measured along the first 4 cm of the LAD (left) and RCA (right) for the 35 suspected-CAD patients, calculated on three reconstructions of prospective low-resolution data (small-k-space NR-PROST followed by bicubic interpolation, padded NR-PROST and the proposed Super-MoCo-MoDL approach) and an NR-PROST reconstruction of the high-resolution acquisition. ‘ns’ denotes a non-significant difference, all other pairwise comparisons exhibited a statistically-significant difference (*p* < 0.0083 following Bonferroni correction for 6 comparisons). (c)-(d) Bland-Altman plots comparing the quantitative sharpness scores of Super-MoCo-MoDL with the high-resolution images; no significant difference is observed for either artery. *CAD* coronary artery disease, *LAD* left anterior descending artery, *NR-PROST* non-rigid motion-corrected patch-based low-rank reconstruction method, *RCA* right coronary artery, *SR* super resolution

Results of the quantitative vessel sharpness analysis are presented in Fig. 11. In Fig. 11 (a), an example reformatted plane depicting the RCA and LAD is shown for the NR-PROST reconstruction (on zero-padded k-space), the Super-MoCo-MoDL reconstruction and the high-resolution (NR-PROST) image. A qualitative improvement in sharpness is evident in this example, with comparable sharpness observed in the Super-MoCo-MoDL and high-resolution images. For both the LAD and RCA, a statistically significant increase in mean sharpness across the 35 suspected-CAD patients is observed for Super-MoCo-MoDL (LAD: 52 ± 8%, RCA: 55 ± 11%) and relative to the NR-PROST reconstructions of low-resolution data (LAD: 49 ± 9%, RCA: 49 ± 12%, padded NR-PROST). Additionally, the Super-MoCo-MoDL scores suggest the method achieves an equivalent level of sharpness to the high-resolution NR-PROST images, with no statistically significant difference observed (LAD: *p* = 0.25, RCA: *p* = 0.013). We note that the *p*-value for the RCA was not considered significant due to the Bonferroni correction for 6 pair-wise comparisons, as outlined in Section 2.8, but that in this instance the sharpness achieved by Super-MoCo-MoDL (55 ± 11%) was greater than that achieved by the high-resolution acquisition (52 ± 11%).

## 4 Discussion

In this work, we have proposed the Super-MoCo-MoDL framework, which combines super-resolution with a motion-corrected model-based deep-learning reconstruction. The proposed technique leverages the advantages afforded by explicitly incorporating data consistency into image reconstruction, including increased robustness and reduction in the requisite amount of training data [93], [94]. While this strategy is often employed in unrolled algorithms for undersampled CMR reconstruction, it is not typically utilized in super-resolution algorithms.

The Super-MoCo-MoDL framework was tested on 15 retrospectively down-sampled scans and applied to 5 prospective low-resolution CHD-patient scans (acquired with 4 × 4 SR and approximately full sampling) and 35 prospective suspected-CAD patient scans (acquired with 2 × 2 SR and 4.5-fold undersampling).

Reconstructed images, expert image-quality scores and rankings and quantitative vessel-sharpness measurements for prospective low-resolution scans all suggested Super-MoCo-MoDL achieves image quality and sharpness approaching that of prospective high-resolution acquisitions, with the images, image rankings and vessel sharpness measurements additionally demonstrating increased sharpness relative to NR-PROST reconstructions of the same low-resolution data.

In all suspected-CAD patients, stenosis, where seen on the 0.9-mm isotropic scan, was also visualized on the Super-MoCo-MoDL reconstruction scan. In some cases, the plaque was more clearly depicted, or had a more similar visual appearance to the CTCA, than the 0.9-mm isotropic acquisition. These improvements were most notable in areas where signal-to-noise limitations in the 0.9-mm images affected visualization. Additionally, since the scan time of the proposed Super-MoCo-MoDL approach is significantly reduced relative to the high-resolution scan, Super-MoCo-MoDL is likely to reduce the risk of patient discomfort and the introduction of bulk motion artifacts. However, there were instances where coronary plaque was better visualized on the 0.9-mm isotropic images than with Super-MoCo-MoDL. These observations suggest that while the proposed approach can enhance image quality and facilitate improved depiction of coronary anatomy, its performance on plaque detection may vary depending on lesion characteristics and anatomical context. Improvements may be made by increasing the acquired spatial resolution of the super-resolution image. Larger scale validation studies are warranted to ensure consistent diagnostic reliability.

When the four images for each patient were ranked from best to worst, the Super-MoCo-MoDL image was selected as best most often (for 23 patients), followed by the high-resolution reference (12 patients). Notably, the Super-MoCo-MoDl reconstruction was never ranked last and was ranked third for only one patient. Conversely, the expert image-quality scores for the suspected-CAD patients (Fig. 10) did not suggest that Super-MoCo-MoDL achieved improved quality, sharpness or robustness to artifacts, relative to the NR-PROST reconstructions. However, they also did not suggest that the high-resolution reference images were superior to the NR-PROST low-resolution images. We hypothesize that this may be due to the broad categories of the Likert scale which see many images awarded the same score.

To confirm the effectiveness of integrating non-rigid motion estimation and correction within the iterative reconstruction, the framework was also trained with the 2 × 2 SR scheme using identical parameters to the previous training except that the non-rigid motion warping operation was skipped during each data consistency step of the MoDL iteration. As previously, iNAV-based translational motion correction was applied directly to the k-space. A comparison of the results achieved when applying the framework, with and without non-rigid motion correction, to the 2 × 2 SR retrospective test set, is included in Supplementary File: Section 8. Example images suggest that small details are more clearly delineated when non-rigid motion is included. Additionally, non-rigid motion correction is seen to result in a statistically significant decrease in MSE ([1.94 ± 0.63] × 10^−3^ vs [1.51 ± 0.50] × 10^−3^]) and a statistically significant increase in SSIM (0.77 ± 0.03 vs 0.80 ± 0.03).

The quantitative error metrics evaluated on retrospectively down-sampled data suggest the 2 × 2 SR scheme, which combines super resolution with undersampling, outperforms the 4 × 4 scheme. Given that both schemes exhibit approximately equal overall acceleration, the advantage of the 2 × 2 scheme may arise as a benefit of distributing the reconstruction across SR and undersampling tasks, alongside the presence of higher frequency k-space lines (relative to the highest frequency acquired under the 4 × 4 scheme). This trend was also reflected in the prospective reconstructions. While the comparison is limited by the differing image resolutions, patient cohorts and samples sizes, we note that the mean overall quality score increased for every reconstruction method when comparing 4 × 4 SR with 2 × 2 SR (small k-space NR-PROST: 1.2 to 3.25; padded NR-PROST: 1 to 3.1; Super-MoCo-MoDL: 2.8 to 3.5) These increases were larger than that seen in the corresponding high-resolution images (3.4 to 3.6), which represents the increase that might be attributed to the underlying acquisition parameters as opposed to the chosen SR scheme.

For the reconstructions of retrospective data, the lack of any significant improvement in MSE or SSIM relative to padded NR-PROST may, in part, be due to the fact that these metrics are not perfect measures of image quality as perceived by human observers. It was for this reason that a VGG-19 [89] perceptual loss function was chosen as the loss on the reconstructed image in Eq. (16) [88]. Had MSE, SSIM or both been used in the training objective instead, the resultant MSE and SSIM figures for the Super-MoCo-MoDL reconstructions would likely have been improved, at the cost of less-sharp images with lower perceived quality. Additionally, since fully sampled high-resolution data were not available to reconstruct ground-truth images against which the low-resolution reconstructions could be measured, the high-resolution reference images were instead formed via NR-PROST reconstructions of 3- or 4-fold undersampled high-resolution data. Thus, any potential bias introduced by the NR-PROST reconstruction, such as the removal of genuine image information alongside noise when applying patch-based low-rank thresholding, may be shared between the padded NR-PROST reconstructions of low-resolution data and the reference image they are being compared against.

The artifacts observed in the 4 × 4-SR Super-MoCo-MoDL reconstructions, which appear as vertical-line patterns in the coronal and sagittal views and as a pixellation-like pattern in the axial view, are aligned with the fully sampled *k*_*x*_ readouts, and thus correspond to unacquired high-frequency components located in the outer region of the *k*_*y*_*k*_*z*_-plane. Their appearance could potentially be suppressed by including additional loss terms in the end-to-end training objective given in Eq. (16) such as, for instance, a spatial smoothness loss, which is not currently explicitly incorporated into the training, or a penalty on large-magnitude high-frequency k-space samples. We note, however, that these artifacts are far less evident when using the 2 × 2 SR scheme, which in general provides increased image quality and sharpness in conjunction with Super-MoCo-MoDL.

## 5 Limitations

A limitation of this study is the relatively small number of prospective low-resolution test subjects acquired with the 4 × 4 SR scheme, limiting the effectiveness of a comparison between the 4 × 4 and 2 × 2 schemes with prospective acquisitions. Additionally, since the patient cohorts and target resolutions for the two schemes were different (4 × 4: CHD, 1.5 mm; 2 × 2: Suspected CAD, 0.9 mm), direct comparisons could only be made using the retrospectively down-sampled data.

An additional limitation is that, although the target SR factor and sampling density profile was carefully matched with the prospective data for training data generation, the target resolution for the suspected-CAD patients (0.9 mm isotropic) was higher than that of any data seen during training. While the SR U-Net is expected to learn the relationship between low- and high-resolution images according to the relative increase in resolution, if this relationship is not the same at different image resolutions, due, for instance, to the enhanced visualization of small-scale anatomical features, the mismatch in resolutions could result in reduced performance. Future work could evaluate the network as trained on higher-resolution data.

While maintaining the same overall undersampling factor, many different combinations of SR factor and degree of VD-CASPR undersampling are possible, including non-integer SR factors. In this study, we have hypothesized that improved reconstruction performance may result from combining SR with undersampling, but no tests were conducted on the optimal degree of SR vs undersampling.

Evaluating the generalizability of the proposed approach to differing undersampling patterns, target resolutions, magnetic field strengths and to sequences with differing image contrast or to simultaneous multi-contrast sequences, remains a topic for future work.

## 6 Conclusion

We have proposed Super-MoCo-MoDL, a deep-learning reconstruction technique for whole-heart CMR that combines data-consistent super-resolution with a motion-corrected model-based iterative reconstruction. The framework was evaluated on a retrospective test set and then applied to prospective low-resolution scans of CHD patients and to prospective undersampled low-resolution scans of patients with suspect CAD. Whole-heart 3D images at 1.5-mm and 0.9-mm isotropic resolution were reconstructed from ∼ 0.8-minute and ∼ 2.1-minute scans, respectively, representing an overall undersampling factor of ∼ 18. Expert image quality scoring and ranking and quantitative vessel sharpness measurements demonstrated that the method achieves comparable quality and sharpness to prospective high-resolution acquisitions, while comparisons with CTCA and high-resolution CMR images for patients with CAD confirmed that Super-MoCo-MoDL performed comparably for the detection of significant coronary stenosis.

## Funding

The authors acknowledge financial support from: (1) BHF programme grant RG/20/1/34802 and King’s BHF Centre for Research Excellence RE/24/130035, (2) Millennium Institute for Intelligent Healthcare Engineering ICN2021 004, (3) Fondecyt 1250261, Fondecyt 1250252, (4) IMPACT, Center of Interventional Medicine for Precision and Advanced Cellular Therapy FB210024, (5) the Department of Health through the National Institute for Health Research (NIHR) comprehensive Biomedical Research Centre award, (6) NIHR Cardiovascular MedTech Co-operative, (7) the Technical University of Munich - Institute for Advanced Study, (8) health insurance “danmark” 2020–0106 and (9) the 10.13039/501100004046Karen Elise Jensen Foundation.

## Author contributions

**Andrew Phair:** Writing – review & editing, Writing – original draft, Visualization, Software, Methodology, Investigation, Formal analysis, Data curation, Conceptualization. **Simon J. Littlewood:** Writing – review & editing, Writing – original draft, Visualization, Investigation, Formal analysis, Data curation. **Anastasia Fotaki:** Writing – review & editing, Investigation, Data curation. **Thomas J. Fletcher:** Writing – review & editing, Software, Methodology. **Lina Felsner:** Writing – review & editing, Software, Methodology, Conceptualization. **Won-Yong Kim:** Writing – review & editing, Funding acquisition. **Claudia Prieto:** Writing – review & editing, Supervision, Resources, Funding acquisition, Conceptualization. **René Botnar:** Writing – review & editing, Supervision, Resources, Funding acquisition, Conceptualization.

## Declaration of competing interests

The authors declare that they have no known competing financial interests or personal relationships that could have appeared to influence the work reported in this paper.

## Data Availability

The datasets generated and analyzed in this study are available from the authors upon reasonable request.
