# Supplementary material for: Super‐MoCo‐MoDL: A combined super-resolution and motion-corrected undersampled deep-learning reconstruction framework for three-dimensional whole-heart cardiac magnetic resonance imaging
Source: J Cardiovasc Magn Reson. 2025 Nov 19;28(1):101990. doi: 10.1016/j.jocmr.2025.101990 (PMC12805380; doi:10.1016/j.jocmr.2025.101990)
Supplement: Supplementary file 1 — Supplementary material [file mmc1.pdf]

Super-MoCo-MoDL: A combined super-resolution and motion-corrected undersampled deep-learning reconstruction framework for 3D whole-heart cardiac MRI

Supplementary Material

Andrew Phair, Simon J. Littlewood, Anastasia Fotaki, Thomas J. Fletcher, Lina Felsner, Won-Yong Kim, Claudia Prieto and René Botnar

1. Acquisition Diagram

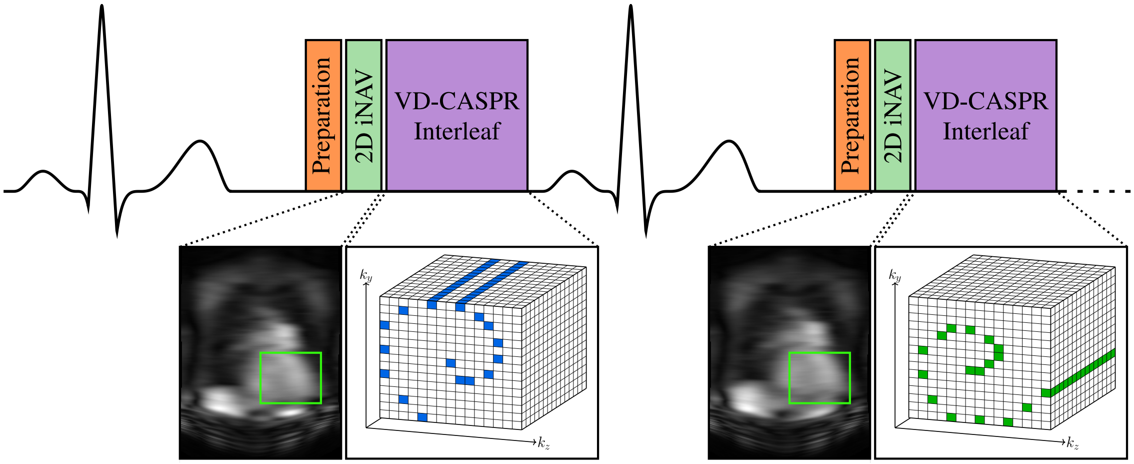

Figure 1: 3D ECG-triggered bSSFP acquisition sequences with data acquired on a VD-CASPR spiral-like Cartesian trajectory. A low-resolution 2D iNAV was acquired at each heartbeat for respiratory motion binning. Preparation pulse(s) varied between the single-contrast sequence, the MTC-BOOST sequence and the iT$_2$-prep-BOOST sequence. 2D - two-dimensional; 3D - three-dimensional; BOOST - bright-blood and black-blood phase sensitive; bSSFP - balanced steady-state free precession; ECG - electrocardiogram; iNAV - image navigator; iT_2-prep - interleaved T_2-preparation; MTC - magnetisation transfer contrast; VD-CASPR - variable density Cartesian acquisition with spiral profile order.

2. Network Diagrams

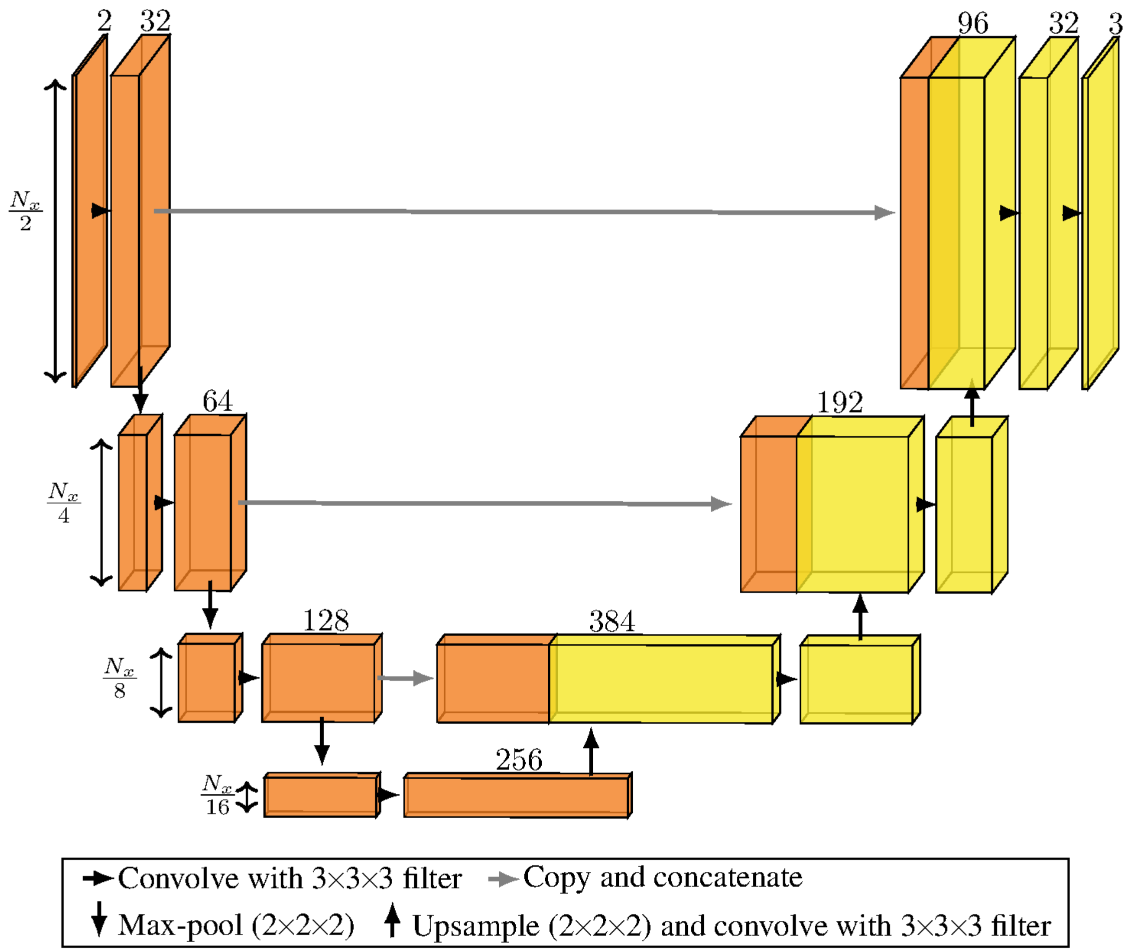

Figure 2: Architecture of the DiRespME-Net. The network takes as input a pair of 3D magnitude images (or image patches) and outputs a 3D velocity field. The velocity field subsequently passes through a scaling and squaring process to produce a diffeomorphic motion field. The y and z image dimensions scale with the x dimension, depicted explicitly, at each layer of the network. 3D - three-dimensional; DiRespME-Net – diffeomorphic respiratory motion estimation network.

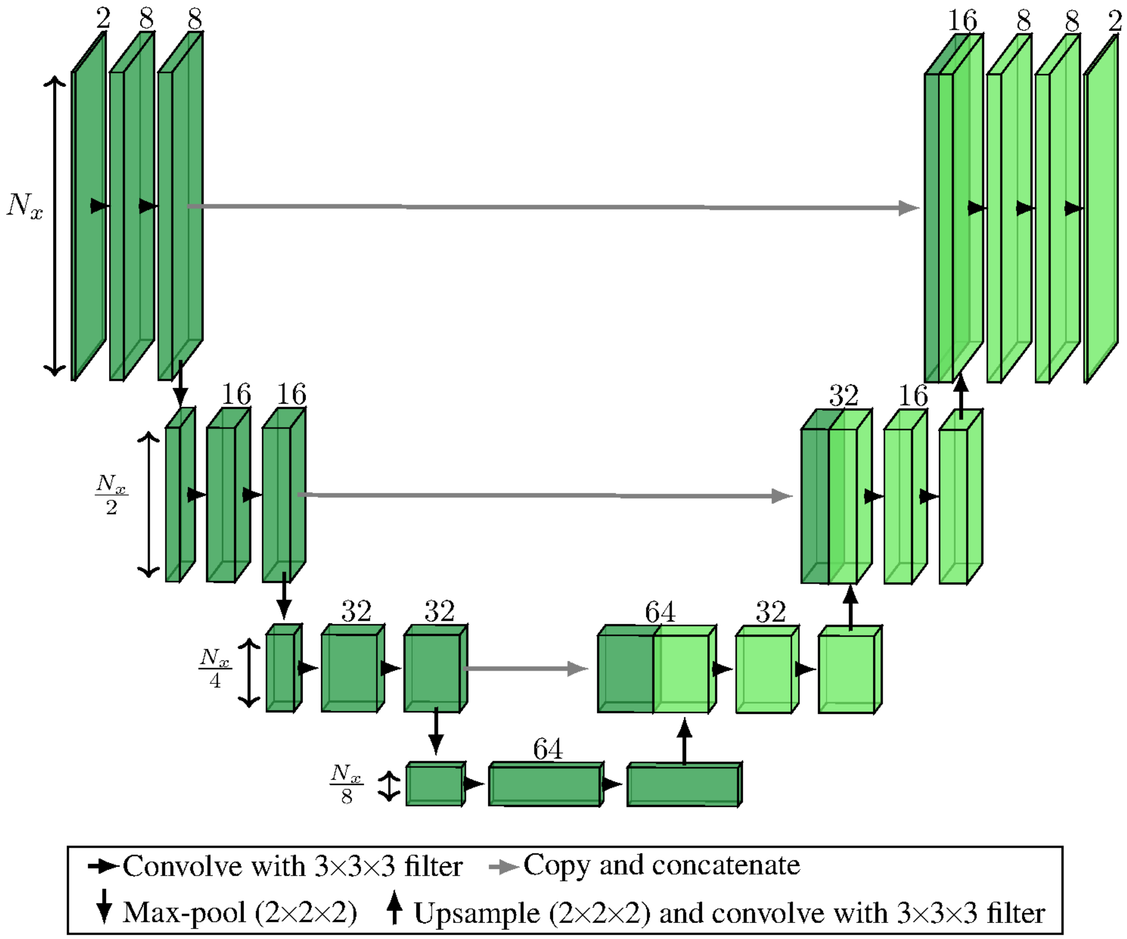

Figure 3: Architecture of the super-resolving U-Net. The network takes as input a low-resolution and undersampled image (or image patches), with the real and complex components input as separate channels, and outputs an image of the same array size. The y and z image dimensions scale with the x dimension, depicted explicitly, at each layer of the network.

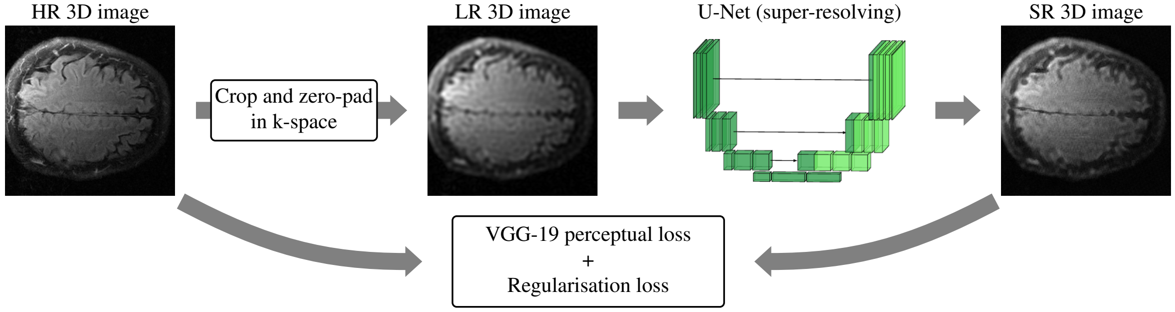

Figure 4: Pre-training of the SR U-Net. Retrospectively down-sampled 3D images are input to the U-Net, and a perceptual loss is calculated between the output image and the high-resolution reference image. 3D - three-dimensional; SR – super resolution.

3. Derivation of ADMM Step 2 Implementation

To implement Step 2 of the ADMM scheme, we must minimise the objective

$$F(\boldsymbol{\omega}) = \frac{\mu}{2}\left\|\boldsymbol{\omega} - S(\boldsymbol{\omega})\right\|_2^2 + \frac{\lambda}{2}\left\|\boldsymbol{\omega} - \tilde{\boldsymbol{\omega}}\right\|_2^2 \quad (1)$$

given the assumption that S is such that

$$\underset{\mathbf{d}}{\operatorname{argmin}}\left\{\|\mathbf{t} - S(\mathbf{d})\|_2^2\right\} = \mathbf{t}. \quad (2)$$

To do so, we consider the N-dimension Euclidean hyperspace that the N-voxel images exist in. Any possible N-voxel image, including any solution that minimises (1), is uniquely defined as a point in this hyperspace.

3.1. Sub-result 1

Firstly, we show by contradiction that for any point $\boldsymbol{\alpha}$ that lies on the straight line between $\tilde{\boldsymbol{\omega}}$ and $S(\tilde{\boldsymbol{\omega}})$, including the end-points, $S(\boldsymbol{\alpha}) = S(\tilde{\boldsymbol{\omega}})$.

Suppose $S(\boldsymbol{\alpha}) \neq S(\tilde{\boldsymbol{\omega}})$, as depicted in Fig. 5. Applying the triangle inequality to the triangle formed by the points $\tilde{\boldsymbol{\omega}}$, $\boldsymbol{\alpha}$ and $S(\boldsymbol{\alpha})$, we have

$$\|\tilde{\boldsymbol{\omega}} - S(\boldsymbol{\alpha})\|_2 < \|\boldsymbol{\alpha} - S(\boldsymbol{\alpha})\|_2 + \|\tilde{\boldsymbol{\omega}} - \boldsymbol{\alpha}\|_2. \quad (3)$$

Here, we have ignored the degenerate triangle case where the two sides of (3) are equal, since we are supposing $S(\boldsymbol{\alpha}) \neq S(\tilde{\boldsymbol{\omega}})$. Now, Eq. (2) implies that

$$\|\boldsymbol{\alpha} - S(\boldsymbol{\alpha})\|_2^2 \leq \|\boldsymbol{\alpha} - S(\tilde{\boldsymbol{\omega}})\|_2^2 \quad (4)$$

and thus

$$\|\boldsymbol{\alpha} - S(\boldsymbol{\alpha})\|_2 \leq \|\boldsymbol{\alpha} - S(\tilde{\boldsymbol{\omega}})\|_2. \quad (5)$$

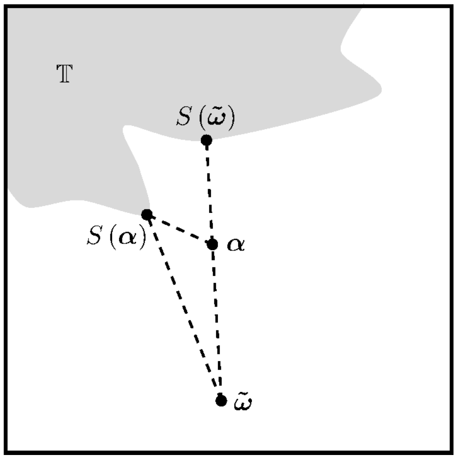

Figure 5: Diagram depicting the scenario where a point $\boldsymbol{\alpha}$ lies on the straight line between $\tilde{\boldsymbol{\omega}}$ and $S(\tilde{\boldsymbol{\omega}})$, but $S(\boldsymbol{\alpha}) \neq S(\tilde{\boldsymbol{\omega}})$. A contradiction arises in this scenario.

Adding $\|\tilde{\boldsymbol{\omega}} - \boldsymbol{\alpha}\|_2$ to (5) yields

$$\|\boldsymbol{\alpha} - S(\boldsymbol{\alpha})\|_2 + \|\tilde{\boldsymbol{\omega}} - \boldsymbol{\alpha}\|_2 \leq \|\boldsymbol{\alpha} - S(\tilde{\boldsymbol{\omega}})\|_2 + \|\tilde{\boldsymbol{\omega}} - \boldsymbol{\alpha}\|_2, \quad (6)$$

which can be combined with (3) to give

$$\|\tilde{\boldsymbol{\omega}} - S(\boldsymbol{\alpha})\|_2 < \|\boldsymbol{\alpha} - S(\tilde{\boldsymbol{\omega}})\|_2 + \|\tilde{\boldsymbol{\omega}} - \boldsymbol{\alpha}\|_2. \quad (7)$$

Noting that the distance between $\tilde{\boldsymbol{\omega}}$ and $S(\tilde{\boldsymbol{\omega}})$ may be expressed as the sum of the two parts of that distance when it is divided at $\boldsymbol{\alpha}$, we also have

$$\|\tilde{\boldsymbol{\omega}} - S(\tilde{\boldsymbol{\omega}})\|_2 = \|\boldsymbol{\alpha} - S(\tilde{\boldsymbol{\omega}})\|_2 + \|\tilde{\boldsymbol{\omega}} - \boldsymbol{\alpha}\|_2. \quad (8)$$

Substituting (8) into (7) gives

$$\|\tilde{\boldsymbol{\omega}} - S(\boldsymbol{\alpha})\|_2 < \|\tilde{\boldsymbol{\omega}} - S(\tilde{\boldsymbol{\omega}})\|_2, \quad (9)$$

which contradicts (2), since it shows $S(\tilde{\boldsymbol{\omega}})$ is not the closest point to $\tilde{\boldsymbol{\omega}}$ in $\mathbb{T}$. Thus, by contradiction, it must be that for any point $\boldsymbol{\alpha}$ which lies on the straight line between $\tilde{\boldsymbol{\omega}}$ and $S(\tilde{\boldsymbol{\omega}})$ (including the end-points), $S(\boldsymbol{\alpha}) = S(\tilde{\boldsymbol{\omega}})$.

3.2. Sub-result 2

Next, we show by contradiction that there must be a solution that minimises the objective in (1) lying on the straight line that passes through $\tilde{\boldsymbol{\omega}}$ and $S(\tilde{\boldsymbol{\omega}})$ and, further, that it must lie on or between those two points.

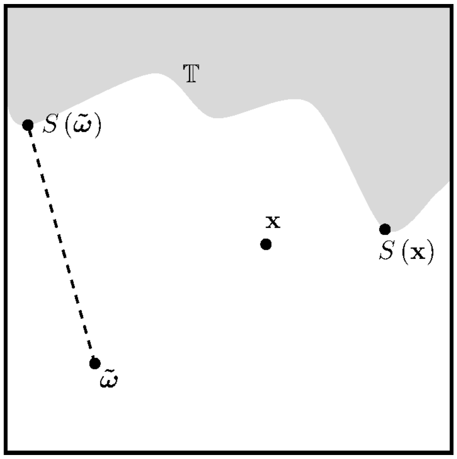

Figure 6: Diagram depicting the scenario where a solution $\mathbf{x}$ does not lie on the straight line between $\tilde{\boldsymbol{\omega}}$ and $S(\tilde{\boldsymbol{\omega}})$.

Suppose a solution $\mathbf{x}$ exists that does not lie on the straight line between the points $\tilde{\boldsymbol{\omega}}$ or $S(\tilde{\boldsymbol{\omega}})$, or on either point, as shown in Fig. 6, and that, if the solution is not unique, no additional solution lies on the line between or on these points.
Let $a = \|\mathbf{x} - S(\mathbf{x})\|_2$ be the distance between $\mathbf{x}$ and $S(\mathbf{x})$, noting that $S(\mathbf{x})$ and $S(\tilde{\boldsymbol{\omega}})$ need not be unique points.
We also define a point $\mathbf{y}$ as

$$\mathbf{y} = S(\tilde{\boldsymbol{\omega}}) + a\frac{\tilde{\boldsymbol{\omega}} - S(\tilde{\boldsymbol{\omega}})}{\|\tilde{\boldsymbol{\omega}} - S(\tilde{\boldsymbol{\omega}})\|_2}, \tag{10}$$

the point at a distance of a from $S(\tilde{\boldsymbol{\omega}})$ in the direction towards $\tilde{\boldsymbol{\omega}}$, as shown in Fig. 7. Since $a \geq 0$, there are now two cases to consider:

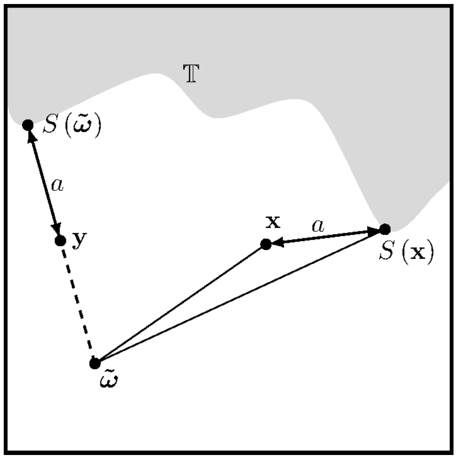

Figure 7: Diagram depicting the scenario where a solution $\mathbf{x}$ does not lie on the straight line between $\tilde{\boldsymbol{\omega}}$ and $S(\tilde{\boldsymbol{\omega}})$ and an additional point $\mathbf{y}$ has been defined at a distance a from $S(\tilde{\boldsymbol{\omega}})$ in the direction towards $\tilde{\boldsymbol{\omega}}$.

1. $\mathbf{y} = S(\tilde{\boldsymbol{\omega}})$, $\mathbf{y}$ lies between $S(\tilde{\boldsymbol{\omega}})$ and $\tilde{\boldsymbol{\omega}}$, or $\mathbf{y} = \tilde{\boldsymbol{\omega}}$; or

2. $\mathbf{y}$ lies beyond $\tilde{\boldsymbol{\omega}}$, as depicted in Fig. 8.

3.2.1 Case 1

Applying the triangle inequality to the triangle formed by the points $\mathbf{x}$, $S(\mathbf{x})$ and $\tilde{\boldsymbol{\omega}}$ gives

$$\|\tilde{\boldsymbol{\omega}} - S(\mathbf{x})\|_2 \leq \|\tilde{\boldsymbol{\omega}} - \mathbf{x}\|_2 + a. \tag{11}$$

Eq. (2) implies that

$$\|\tilde{\boldsymbol{\omega}} - S(\tilde{\boldsymbol{\omega}})\|_2^2 \leq \|\tilde{\boldsymbol{\omega}} - S(\mathbf{x})\|_2^2 \tag{12}$$

and thus

$$\|\tilde{\boldsymbol{\omega}} - S(\tilde{\boldsymbol{\omega}})\|_2 \leq \|\tilde{\boldsymbol{\omega}} - S(\mathbf{x})\|_2. \tag{13}$$

Now, combining (11) and (13) gives

$$\|\tilde{\boldsymbol{\omega}} - S(\tilde{\boldsymbol{\omega}})\|_2 \leq \|\tilde{\boldsymbol{\omega}} - \mathbf{x}\|_2 + a. \tag{14}$$

Since, in this case, $\mathbf{y}$ lies on or between $S(\tilde{\boldsymbol{\omega}})$ and $\tilde{\boldsymbol{\omega}}$, the distance between the two, $\|\tilde{\boldsymbol{\omega}} - S(\tilde{\boldsymbol{\omega}})\|_2$ may be expressed as the sum of the two parts of that distance when it is divided at $\mathbf{y}$:

$$\|\tilde{\boldsymbol{\omega}} - S(\tilde{\boldsymbol{\omega}})\|_2 = \|\tilde{\boldsymbol{\omega}} - \mathbf{y}\|_2 + a. \tag{15}$$

Substituting (15) into (14), subtracting a and squaring both sides gives

$$\|\tilde{\boldsymbol{\omega}} - \mathbf{y}\|_2^2 \leq \|\tilde{\boldsymbol{\omega}} - \mathbf{x}\|_2^2 . \tag{16}$$

Since the penalty weighting parameters λ and μ are non-negative, multiplying (16) by $\frac{\lambda}{2}$ and adding $\frac{\mu}{2}a^2$ yields

$$\frac{\mu}{2}a^2 + \frac{\lambda}{2}\|\tilde{\boldsymbol{\omega}} - \mathbf{y}\|_2^2 \leq \frac{\mu}{2}a^2 + \frac{\lambda}{2}\|\tilde{\boldsymbol{\omega}} - \mathbf{x}\|_2^2 . \tag{17}$$

Substituting in the equivalent expressions $a = \|\mathbf{y} - S(\tilde{\boldsymbol{\omega}})\|_2$ and $a = \|\mathbf{x} - S(\mathbf{x})\|_2$ to the left and right sides of the inequality, respectively, we find

$$\begin{aligned} &\frac{\mu}{2}\|\mathbf{y} - S(\tilde{\boldsymbol{\omega}})\|_2^2 + \frac{\lambda}{2}\|\tilde{\boldsymbol{\omega}} - \mathbf{y}\|_2^2 \\ &\quad \leq \frac{\mu}{2}\|\mathbf{x} - S(\mathbf{x})\|_2^2 + \frac{\lambda}{2}\|\tilde{\boldsymbol{\omega}} - \mathbf{x}\|_2^2 . \end{aligned} \tag{18}$$

Now, since $\mathbf{y}$ lies on the line between $S(\tilde{\boldsymbol{\omega}})$ and $\tilde{\boldsymbol{\omega}}$, or on the endpoints, sub-result 1 implies that $S(\mathbf{y}) = S(\tilde{\boldsymbol{\omega}})$. Substituting this into (18) gives

$$\begin{aligned} &\frac{\mu}{2}\|\mathbf{y} - S(\mathbf{y})\|_2^2 + \frac{\lambda}{2}\|\tilde{\boldsymbol{\omega}} - \mathbf{y}\|_2^2 \\ &\quad \leq \frac{\mu}{2}\|\mathbf{x} - S(\mathbf{x})\|_2^2 + \frac{\lambda}{2}\|\tilde{\boldsymbol{\omega}} - \mathbf{x}\|_2^2 \end{aligned} \tag{19}$$

which we recognise as an inequality of two expressions in the form of the objective defined in (1), and thus

$$F(\mathbf{y}) \leq F(\mathbf{x}). \tag{20}$$

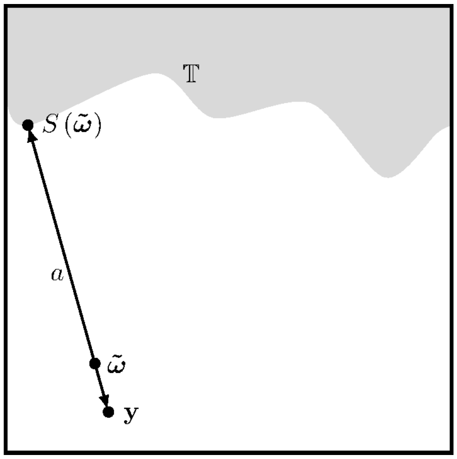

Figure 8: Diagram depicting the scenario where the additional point $\mathbf{y}$ lies beyond $\tilde{\boldsymbol{\omega}}$.

Therefore, either $F(\mathbf{y}) < F(\mathbf{x})$, and thus $\mathbf{x}$ does not minimise F, or $F(\mathbf{y}) = F(\mathbf{x})$, and an additional solution that minimises to (1) does lie on the line between the points $\tilde{\boldsymbol{\omega}}$ and $S(\tilde{\boldsymbol{\omega}})$. In either instance, (20) represents a contradiction of our supposition.

3.2.2 Case 2

When $\mathbf{y}$ lies beyond $\tilde{\boldsymbol{\omega}}$, as depicted in Fig. 8, $a > \|\tilde{\boldsymbol{\omega}} - S(\tilde{\boldsymbol{\omega}})\|_2$, implying

$$\frac{\mu}{2}\|\mathbf{x} - S(\mathbf{x})\|_2^2 > \frac{\mu}{2}\|\tilde{\boldsymbol{\omega}} - S(\tilde{\boldsymbol{\omega}})\|_2^2 , \tag{21}$$

since μ is non-negative. As $\frac{\lambda}{2}\|\mathbf{x} - \tilde{\boldsymbol{\omega}}\|_2^2$ is also non-negative, it may be added to the left-hand side of the inequality in (21) to yield

$$F(\mathbf{x}) > \frac{\mu}{2}\|\tilde{\boldsymbol{\omega}} - S(\tilde{\boldsymbol{\omega}})\|_2^2 . \tag{22}$$

Now, evaluating $F(\tilde{\boldsymbol{\omega}})$ using the definition given by (1), we have

$$\begin{aligned} F(\tilde{\boldsymbol{\omega}}) &= \frac{\mu}{2}\left\|\tilde{\boldsymbol{\omega}} - S(\boldsymbol{\omega})\right\|_2^2 + \frac{\lambda}{2}\left\|\tilde{\boldsymbol{\omega}} - \tilde{\boldsymbol{\omega}}\right\|_2^2 \\ &= \frac{\mu}{2}\left\|\tilde{\boldsymbol{\omega}} - S(\boldsymbol{\omega})\right\|_2^2. \end{aligned} \tag{23}$$

Substituting (23) into (22) gives

$$F(\mathbf{x}) > F(\tilde{\boldsymbol{\omega}}), \tag{24}$$

demonstrating that $\mathbf{x}$ does not minimise F.

In both case 1 and case 2, a contradiction arises. Thus, by contradiction, we have show that there must be a solution that minimises the objective in (1) which *does* lie on the straight line that passes through $\tilde{\boldsymbol{\omega}}$ and $S(\tilde{\boldsymbol{\omega}})$ and, further, it must lie on or between those two points.

3.3. Solution by Scalar Differentiation

Given these two sub-results, we may now express (1) as a function of a scalar variable.
From sub-result 2, the solution lies on the straight line that passes through $\tilde{\boldsymbol{\omega}}$ and $S(\tilde{\boldsymbol{\omega}})$, and hence may be expressed as

$$\boldsymbol{\omega} = \tilde{\boldsymbol{\omega}} + h\left(S(\tilde{\boldsymbol{\omega}}) - \tilde{\boldsymbol{\omega}}\right), \tag{25}$$

and, since it must also lie on or between these points, $h \in [0, 1]$. Additionally, sub-result 1 implies

$$S(\boldsymbol{\omega}) = S(\tilde{\boldsymbol{\omega}}). \tag{26}$$

Substituting (25) and (26) into (1) allows the objective to be expressed as a function of h,

$$\begin{aligned} \hat{F}(h) =& \frac{\mu}{2}\left\|\tilde{\boldsymbol{\omega}} + h\left(S(\tilde{\boldsymbol{\omega}}) - \tilde{\boldsymbol{\omega}}\right) - S(\tilde{\boldsymbol{\omega}})\right\|_2^2 \\ &\quad \ldots + \frac{\lambda}{2}\left\|\tilde{\boldsymbol{\omega}} + h\left(S(\tilde{\boldsymbol{\omega}}) - \tilde{\boldsymbol{\omega}}\right) - \tilde{\boldsymbol{\omega}}\right\|_2^2 \\ =& \frac{\mu}{2}\left\|(h-1)\left(S(\tilde{\boldsymbol{\omega}}) - \tilde{\boldsymbol{\omega}}\right)\right\|_2^2 \\ &\quad \ldots + \frac{\lambda}{2}\left\|h\left(S(\tilde{\boldsymbol{\omega}}) - \tilde{\boldsymbol{\omega}}\right)\right\|_2^2 \\ =& \left[\frac{\mu(h-1)^2}{2} + \frac{\lambda h^2}{2}\right]\left\|S(\tilde{\boldsymbol{\omega}}) - \tilde{\boldsymbol{\omega}}\right\|_2^2, \end{aligned} \tag{27}$$

which has the scalar derivative

$$\hat{F}'(h) = \left[\mu(h-1) + \lambda h\right]\left\|S(\tilde{\boldsymbol{\omega}}) - \tilde{\boldsymbol{\omega}}\right\|_2^2. \tag{28}$$

Since the minimum of $\hat{F}(h)$ occurs when $\hat{F}'(h) = 0$, we set (28) to 0, yielding

$$h = \frac{\mu}{\mu + \lambda}. \tag{29}$$

Finally, substituting (29) into (25), we find

$$\begin{aligned} \boldsymbol{\omega} &= \tilde{\boldsymbol{\omega}} + \left(\frac{\mu}{\mu + \lambda}\right)\left(S(\tilde{\boldsymbol{\omega}}) - \tilde{\boldsymbol{\omega}}\right) \\ &= \left(\frac{\mu}{\mu + \lambda}\right) S(\tilde{\boldsymbol{\omega}}) + \left(\frac{\lambda}{\mu + \lambda}\right)\tilde{\boldsymbol{\omega}}, \end{aligned} \tag{30}$$

which is the solution that minimises (1).

4. Retrospective Down-sampling Schematics

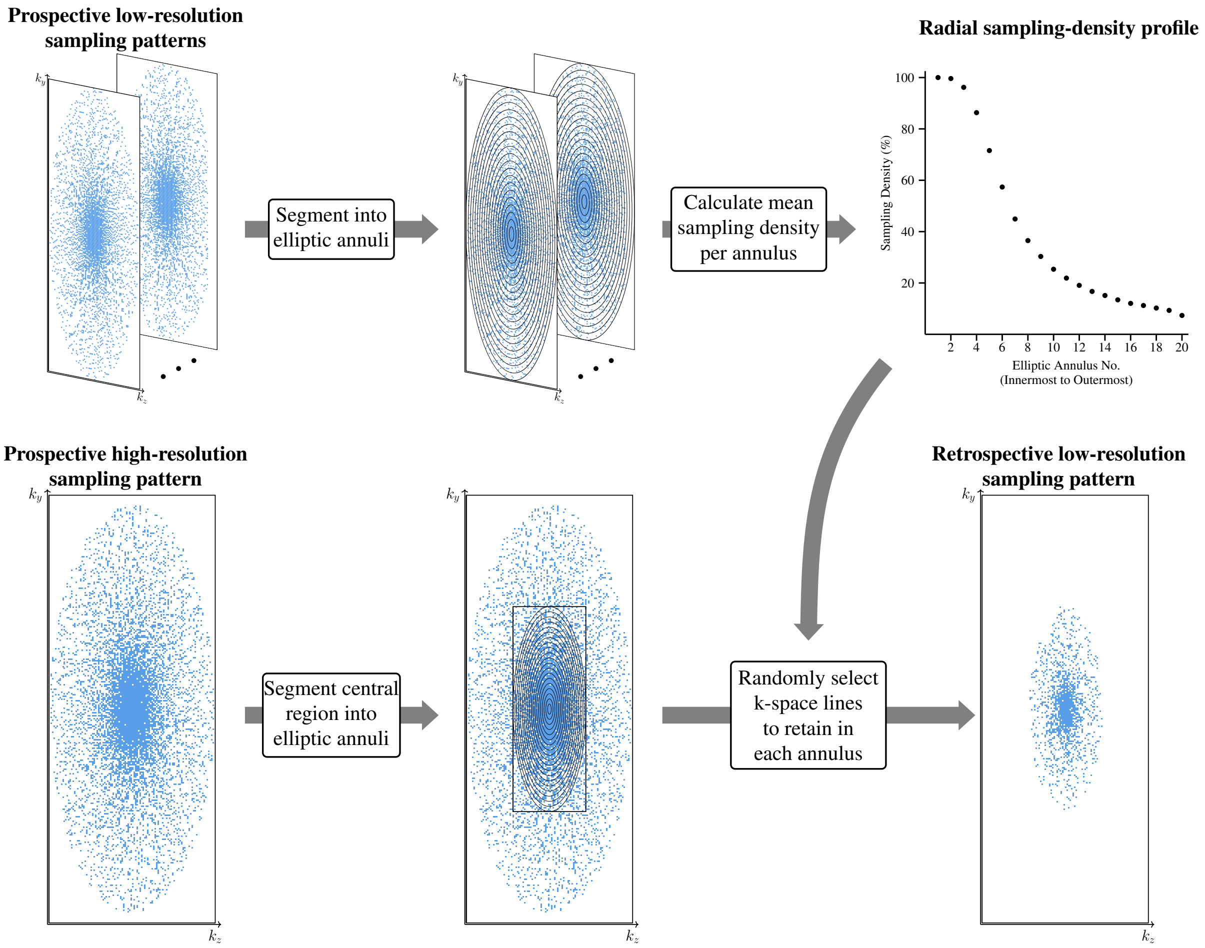

Figure 9: Schematic depicting the retrospective down-sampling procedure for the 2×2 SR scheme with 4.6-fold VD-CASPR undersampling. The sampling density in the k_y-k_z plane is measured across 20 concentric elliptic annuli in prospective acquisitions. The elliptic-annuli segmentation is then overlaid on a high-resolution acquisition at a scale set as half the k-space field of view. In each annulus, acquired k_x readouts are randomly selected to be retained, with the total number retained chosen to ensure the average prospective sampling density in that annulus is matched. SR – super resolution; VD-CASPR - variable density Cartesian acquisition with spiral profile order.

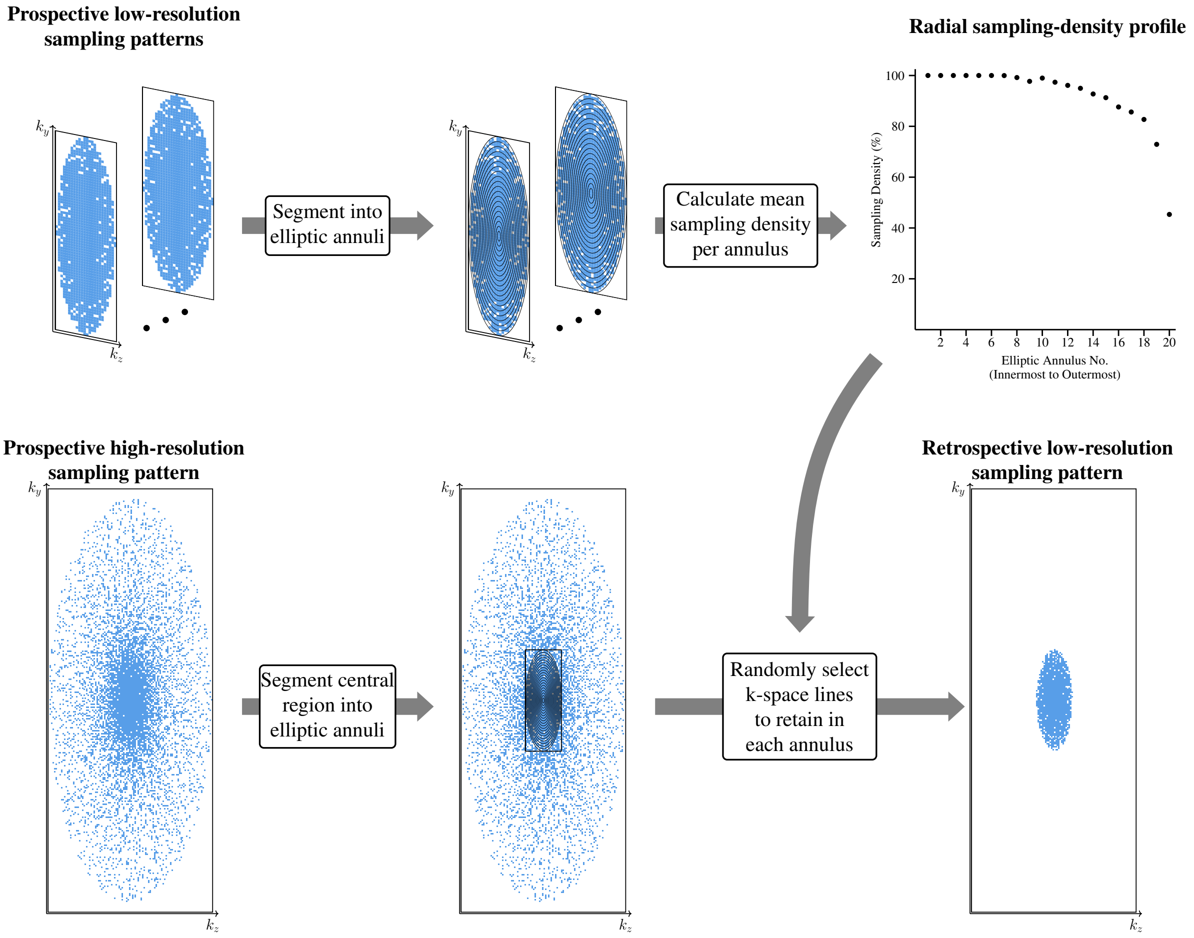

Figure 10: Schematic depicting the retrospective down-sampling procedure for the 4×4 SR scheme with 1.15-fold VD-CASPR undersampling. The sampling density in the k_y-k_z plane is measured across 20 concentric elliptic annuli in prospective acquisitions. The elliptic-annuli segmentation is then overlaid on a high-resolution acquisition at a scale set as a quarter of the k-space field of view. In each annulus, acquired k_x readouts are randomly selected to be retained, with the total number retained chosen to ensure the average prospective sampling density in that annulus is matched. SR – super resolution; VD-CASPR - variable density Cartesian acquisition with spiral profile order.

5. Training-Set and Retrospective-Test-Set Array Sizes

Table 1: Array sizes of training-set images. Highlighted rows indicate those datasets that have been cropped or padded to reduce the number of unique patch sizes seen during training. BOOST - bright-blood and black-blood phase sensitive; iT_2-prep - interleaved T_2-preparation; MTC - magnetisation transfer contrast.

Sequence	No.	Initial Array Size	Rounded Array Size	Final Array Size
Single Contrast	2	272 × 261 × 112	272 × 272 × 112	272 × 272 × 112
Single Contrast	1	272 × 264 × 80	272 × 272 × 80	272 × 272 × 112
Single Contrast	1	272 × 264 × 104	272 × 272 × 112	272 × 272 × 112
Single Contrast	1	272 × 264 × 112	272 × 272 × 112	272 × 272 × 112
Single Contrast	3	272 × 270 × 88	272 × 272 × 96	272 × 272 × 112
Single Contrast	2	272 × 270 × 104	272 × 272 × 112	272 × 272 × 112
Single Contrast	1	272 × 270 × 112	272 × 272 × 112	272 × 272 × 112
Single Contrast	3	272 × 270 × 120	272 × 272 × 128	272 × 272 × 128
Single Contrast	2	272 × 270 × 128	272 × 272 × 128	272 × 272 × 128
Single Contrast	1	272 × 272 × 120	272 × 272 × 128	272 × 272 × 128
Single Contrast	1	272 × 275 × 88	272 × 288 × 96	272 × 288 × 96
Single Contrast	1	272 × 275 × 112	272 × 288 × 112	272 × 288 × 112
Single Contrast	2	272 × 276 × 112	272 × 288 × 112	272 × 288 × 112
Single Contrast	1	272 × 276 × 128	272 × 288 × 128	272 × 288 × 128
Single Contrast	1	272 × 280 × 104	272 × 288 × 112	272 × 288 × 112
Single Contrast	4	272 × 280 × 120	272 × 288 × 128	272 × 288 × 128
Single Contrast	1	272 × 280 × 128	272 × 288 × 128	272 × 288 × 128
Single Contrast	2	272 × 286 × 104	272 × 288 × 112	272 × 288 × 112
Single Contrast	1	272 × 286 × 128	272 × 288 × 128	272 × 288 × 128
Single Contrast	1	272 × 288 × 112	272 × 288 × 112	272 × 288 × 112
Single Contrast	1	272 × 294 × 120	272 × 304 × 128	272 × 304 × 128
Single Contrast	1	272 × 300 × 104	272 × 304 × 112	272 × 304 × 112
MTC-BOOST	1	208 × 270 × 128	208 × 272 × 128	208 × 272 × 128
MTC-BOOST	2	208 × 272 × 120	208 × 272 × 128	208 × 272 × 128
MTC-BOOST	1	208 × 273 × 128	208 × 288 × 128	208 × 288 × 128
MTC-BOOST	2	208 × 275 × 96	208 × 288 × 96	208 × 288 × 96

Table 1: (continued)

Sequence	No.	Initial Array Size	Rounded Array Size	Final Array Size
MTC-BOOST	1	208 × 275 × 112	208 × 288 × 112	208 × 288 × 112
MTC-BOOST	1	208 × 275 × 120	208 × 288 × 128	208 × 288 × 128
MTC-BOOST	1	208 × 279 × 112	208 × 288 × 112	208 × 288 × 112
MTC-BOOST	1	208 × 279 × 128	208 × 288 × 128	208 × 288 × 128
MTC-BOOST	1	208 × 280 × 96	208 × 288 × 96	208 × 288 × 96
MTC-BOOST	1	208 × 280 × 112	208 × 288 × 112	208 × 288 × 112
MTC-BOOST	7	208 × 280 × 120	208 × 288 × 128	208 × 288 × 128
MTC-BOOST	11	208 × 280 × 128	208 × 288 × 128	208 × 288 × 128
MTC-BOOST	2	208 × 280 × 144	208 × 288 × 144	208 × 304 × 128
MTC-BOOST	1	208 × 286 × 112	208 × 288 × 112	208 × 288 × 112
MTC-BOOST	3	208 × 286 × 128	208 × 288 × 128	208 × 288 × 128
MTC-BOOST	2	208 × 288 × 112	208 × 288 × 112	208 × 288 × 112
MTC-BOOST	1	208 × 288 × 120	208 × 288 × 128	208 × 288 × 128
MTC-BOOST	4	208 × 288 × 128	208 × 288 × 128	208 × 288 × 128
MTC-BOOST	1	208 × 288 × 144	208 × 288 × 144	208 × 304 × 128
MTC-BOOST	2	208 × 290 × 120	208 × 304 × 128	208 × 304 × 128
MTC-BOOST	1	208 × 290 × 144	208 × 304 × 144	208 × 304 × 128
MTC-BOOST	1	208 × 297 × 96	208 × 304 × 96	208 × 304 × 128
MTC-BOOST	4	208 × 297 × 112	208 × 304 × 112	208 × 304 × 112
MTC-BOOST	1	208 × 297 × 120	208 × 304 × 128	208 × 304 × 128
MTC-BOOST	1	208 × 297 × 128	208 × 304 × 128	208 × 304 × 128
MTC-BOOST	11	208 × 300 × 112	208 × 304 × 112	208 × 304 × 112
MTC-BOOST	12	208 × 300 × 120	208 × 304 × 128	208 × 304 × 128
MTC-BOOST	15	208 × 300 × 128	208 × 304 × 128	208 × 304 × 128
MTC-BOOST	2	208 × 300 × 144	208 × 304 × 144	208 × 304 × 128
MTC-BOOST	1	208 × 330 × 144	208 × 336 × 144	208 × 304 × 128
iT_2-prep-BOOST	2	288 × 300 × 112	288 × 304 × 112	288 × 304 × 112
iT_2-prep-BOOST	1	256 × 250 × 112	256 × 256 × 112	256 × 272 × 112
iT_2-prep-BOOST	1	256 × 275 × 112	256 × 272 × 112	256 × 288 × 112
iT_2-prep-BOOST	1	304 × 297 × 120	304 × 304 × 128	304 × 304 × 128
iT_2-prep-BOOST	1	304 × 297 × 128	304 × 304 × 128	304 × 304 × 128
iT_2-prep-BOOST	4	304 × 297 × 144	304 × 304 × 144	304 × 304 × 128
iT_2-prep-BOOST	3	304 × 297 × 160	304 × 304 × 160	304 × 304 × 128

Table 1: (continued)

Sequence	No.	Initial Array Size	Rounded Array Size	Final Array Size
iT_2-prep-BOOST	1	304 × 300 × 160	304 × 304 × 160	304 × 304 × 128
iT_2-prep-BOOST	2	304 × 306 × 120	304 × 320 × 128	304 × 304 × 128
iT_2-prep-BOOST	1	304 × 310 × 112	304 × 320 × 112	304 × 304 × 128
iT_2-prep-BOOST	2	304 × 310 × 120	304 × 320 × 128	304 × 304 × 128
iT_2-prep-BOOST	1	304 × 310 × 144	304 × 320 × 144	304 × 304 × 128
iT_2-prep-BOOST	1	304 × 312 × 128	304 × 320 × 128	304 × 304 × 128
iT_2-prep-BOOST	1	304 × 315 × 224	304 × 320 × 224	304 × 304 × 128
iT_2-prep-BOOST	1	304 × 319 × 160	304 × 320 × 160	304 × 304 × 128
iT_2-prep-BOOST	1	304 × 319 × 176	304 × 320 × 176	304 × 304 × 128
iT_2-prep-BOOST	1	304 × 320 × 144	304 × 320 × 144	304 × 304 × 128
iT_2-prep-BOOST	1	304 × 333 × 112	304 × 336 × 112	304 × 304 × 128
iT_2-prep-BOOST	1	304 × 336 × 120	304 × 336 × 128	304 × 304 × 128

Table 2: Array sizes of retrospective-test-set images BOOST - bright-blood and black-blood phase sensitive; iT_2-prep - interleaved T_2-preparation; MTC - magnetisation transfer contrast.

Sequence	No.	Initial Array Size	Rounded Array Size
Single Contrast	1	272 × 261 × 112	272 × 272 × 112
Single Contrast	1	272 × 264 × 80	272 × 272 × 112
Single Contrast	1	272 × 264 × 104	272 × 272 × 112
Single Contrast	1	272 × 264 × 112	272 × 272 × 112
Single Contrast	1	272 × 270 × 88	272 × 272 × 112
MTC-BOOST	1	208 × 270 × 128	208 × 272 × 128
MTC-BOOST	1	208 × 272 × 120	208 × 272 × 128
MTC-BOOST	5	208 × 273 × 128	208 × 288 × 128
MTC-BOOST	2	208 × 275 × 96	208 × 288 × 96
iT_2-Prep-BOOST	1	288 × 300 × 112	288 × 304 × 112

6. Example Images with Varying Overall Image Quality Scores

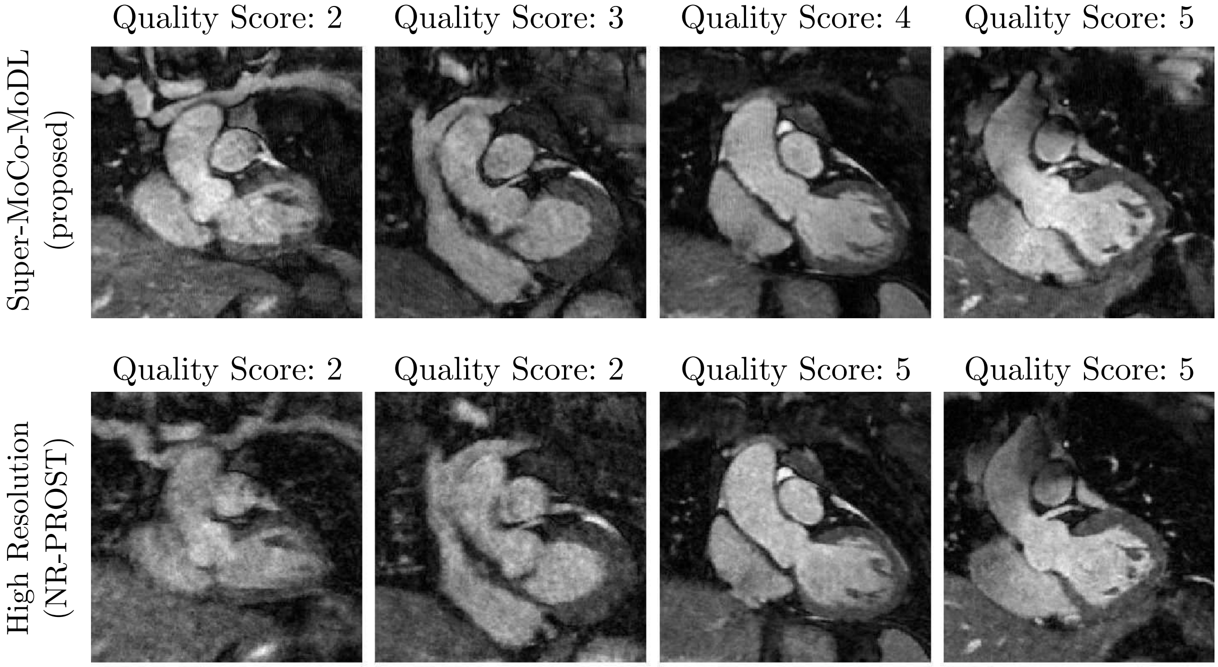

Figure 11: Coronal slices of Super-MoCo-MoDL reconstructions of four prospective low-resolution scans of suspected-CAD patients acquired with the 2×2 SR scheme (top row) and NR-PROST reconstructions of high-resolution acquisitions of the same patients, demonstrating a range of overall image quality scores. Scoring was performed using a 5-point Likert scale defined as: 1 - non-diagnostic; 2 - poor image quality (poor endocardial/vessel wall definitions and/or significant noise/artefact); 3 - adequate image quality (overall sufficient image quality, but one or more structures may be less well-defined or be subject to noise/artefact); 4 - good image quality (structures generally well-defined but may be some noise/artefact); 5 - excellent image quality (sharp definitions of all structures without any significant noise/artefact). No image with score of 1 is included since the minimum score of any Super-MoCo-MoDL reconstruction was 2. Each image is individually normalised. CAD - coronary artery disease; NR-PROST – non-rigid motion-corrected patch-based low-rank reconstruction method.

7. Additional Bland-Altman Plots for Pair-wise Comparison of MSE and SSIM

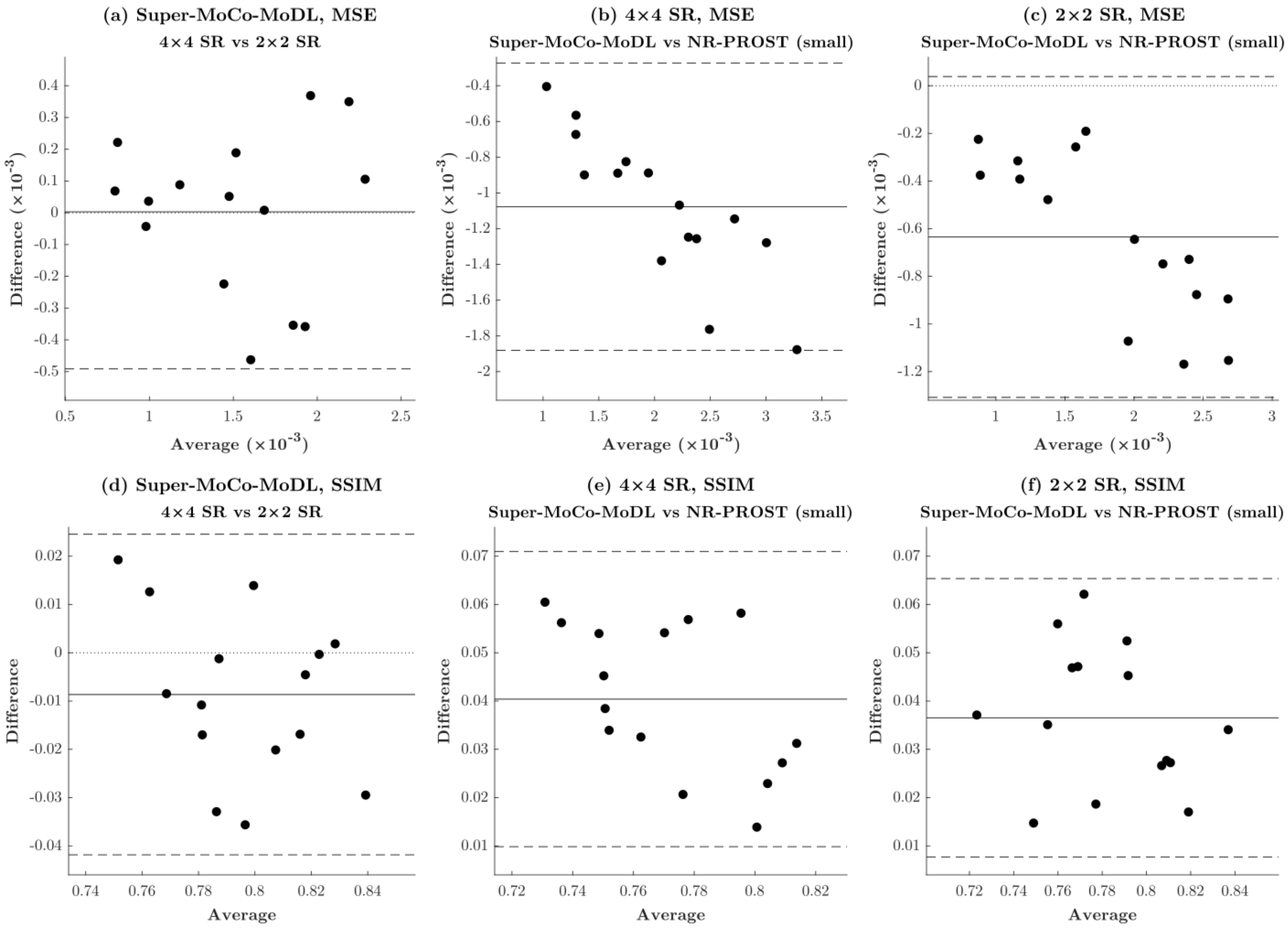

Figure 12: Quantitative error metrics MSE (top) and SSIM (bottom) calculated with the 15-patient retrospective test set relative to NR-PROST reconstructions of high-resolution data. (a) and (d) Comparison of the error measured in Super-MoCo-MoDL reconstructions when applied to data retrospectively downsampled according to the two SR schemes considered. (b)-(c), (e)-(f) Comparison between the error measured in the Super-MoCo-MoDL reconstruction and NR-PROST on a small low-resolution k-space followed by bicubic interpolation, for both the 4×4 ((b), (e)) and 2×2 ((c), (f)) SR schemes. MSE – mean squared error; NR-PROST – non-rigid motion-corrected patch-based low-rank reconstruction method; SR – super resolution; SSIM – structural similarity.

8. Effect of Including Non-rigid Motion Correction in the Framework

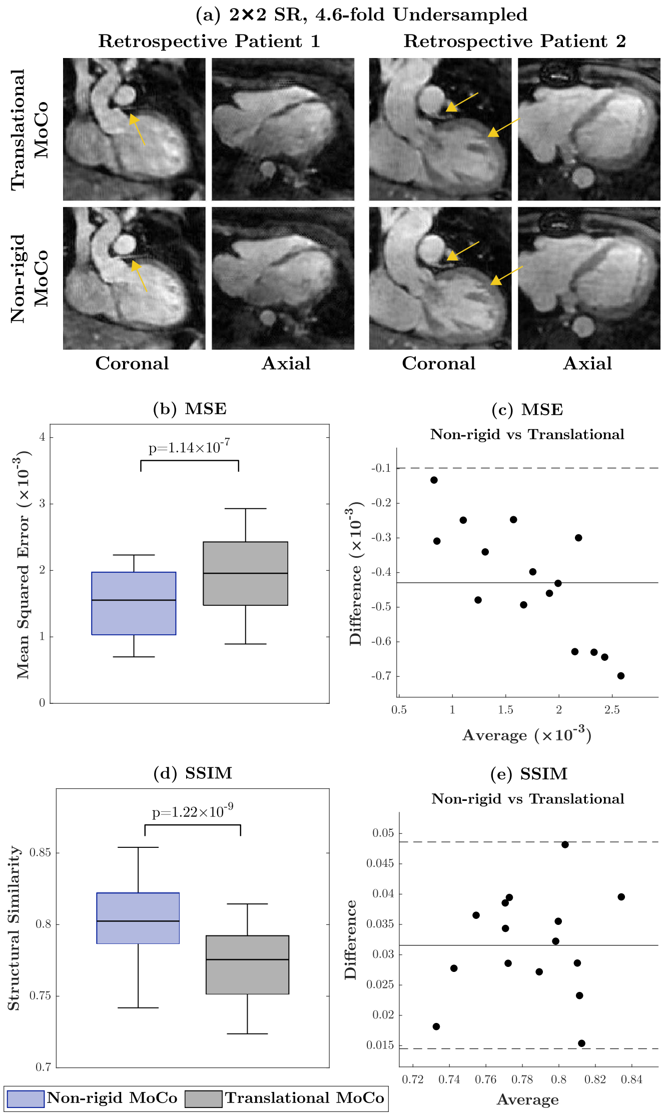

Figure 13: Comparison between Super-MoCo-MoDL reconstructions of data retrospectively undersampled with the 2×2 SR scheme when (i) non-rigid motion correction is included in the iterative reconstruction and (ii) only translational motion correction is applied to the input data. (a) Coronal and axial slices of the reconstructions for two example patients from the retrospective test set. Arrows indicate small features that are better delineated when non-rigid motion correction is incorporated in the reconstruction. (b)-(e) Quantitative error metrics MSE (b)-(c) and SSIM (d)-(e) calculated with the 15-patient retrospective test set relative to NR-PROST reconstructions of high-resolution data. p-values for each metric calculated using a paired-samples t-test are included in the boxplots (b) and (d). MoCo - motion-corrected; MSE – mean squared error; NR-PROST – non-rigid motion-corrected patch-based low-rank reconstruction method; SR – super resolution; SSIM – structural similarity.
